# GADD45A is a mediator of mitochondrial loss, atrophy, and weakness in skeletal muscle

**DOI:** 10.1172/jci.insight.171772

**Published:** 2023-11-22

**Authors:** George R. Marcotte, Matthew J. Miller, Hawley E. Kunz, Zachary C. Ryan, Matthew D. Strub, Patrick M. Vanderboom, Carrie J. Heppelmann, Sarah Chau, Zachary D. Von Ruff, Sean P. Kilroe, Andrew T. McKeen, Jason M. Dierdorff, Jennifer I. Stern, Karl A. Nath, Chad E. Grueter, Vitor A. Lira, Andrew R. Judge, Blake B. Rasmussen, K. Sreekumaran Nair, Ian R. Lanza, Scott M. Ebert, Christopher M. Adams

**Affiliations:** 1 Division of Endocrinology, Diabetes, Metabolism, and Nutrition, Department of Internal Medicine, Mayo Clinic, Rochester, Minnesota, USA.; 2 University of Iowa, Iowa City, Iowa, USA.; 3 University of Texas Medical Branch, Galveston, Texas, USA.; 4 Department of Neurology and; 5 Division of Nephrology and Hypertension, Department of Internal Medicine, Mayo Clinic, Rochester, Minnesota, USA.; 6 University of Florida, Gainesville, Florida, USA.; 7 Emmyon, Inc., Rochester, Minnesota, USA.; 8 University of Texas Health Science Center, San Antonio, Texas, USA.

**Keywords:** Metabolism, Muscle Biology, Mitochondria, Skeletal muscle

## Abstract

Aging and many illnesses and injuries impair skeletal muscle mass and function, but the molecular mechanisms are not well understood. To better understand the mechanisms, we generated and studied transgenic mice with skeletal muscle–specific expression of growth arrest and DNA damage inducible α (GADD45A), a signaling protein whose expression in skeletal muscle rises during aging and a wide range of illnesses and injuries. We found that GADD45A induced several cellular changes that are characteristic of skeletal muscle atrophy, including a reduction in skeletal muscle mitochondria and oxidative capacity, selective atrophy of glycolytic muscle fibers, and paradoxical expression of oxidative myosin heavy chains despite mitochondrial loss. These cellular changes were at least partly mediated by MAP kinase kinase kinase 4, a protein kinase that is directly activated by GADD45A. By inducing these changes, GADD45A decreased the mass of muscles that are enriched in glycolytic fibers, and it impaired strength, specific force, and endurance exercise capacity. Furthermore, as predicted by data from mouse models, we found that *GADD45A* expression in skeletal muscle was associated with muscle weakness in humans. Collectively, these findings identify GADD45A as a mediator of mitochondrial loss, atrophy, and weakness in mouse skeletal muscle and a potential target for muscle weakness in humans.

## Introduction

Skeletal muscle atrophy can be a debilitating consequence of advanced age, malnutrition, muscle disuse, and any severe illness or injury. It touches all areas of medicine and affects most people at some point in their lives. Common and serious effects of muscle atrophy include weakness, fatigue, reduced activity, impaired recovery from illness or injury, frailty, falls, and loss of independent living. Furthermore, by limiting activity, muscle atrophy can be a feed-forward process toward more pronounced muscle loss and functional deficits.

The molecular mechanisms of skeletal muscle atrophy are complex, context dependent, and still not well understood (1–4). Over the past several years, we have investigated the role of activating transcription factor 4 (ATF4), a stress-inducible transcription regulator in the basic leucine zipper (bZIP) superfamily (4). Many conditions that cause muscle atrophy increase ATF4 expression in skeletal muscle (4–6), and a targeted reduction of ATF4 in skeletal muscle fibers reduces muscle atrophy during starvation, immobilization, and aging (6–9). ATF4 promotes muscle atrophy by forming a heterodimer with the liver-enriched activator protein isoform of CCAAT enhancer binding protein β (C/EBPβ), another bZIP transcription regulator (10).

In skeletal muscle fibers, the ATF4-C/EBPβ heterodimer activates several genes, including *Gadd45a* (encoding growth arrest and DNA damage inducible α) (7). The *Gadd45a* gene is weakly expressed in young, healthy skeletal muscle fibers but strongly induced during muscle atrophy (4, 6, 7, 11–23). The ATF4-C/EBPβ heterodimer activates the *Gadd45a* gene in skeletal muscle fibers by binding an ATF4-C/EBPβ response element that is 100% conserved across all available mammalian genomes, including mice and humans (7). In addition to the ATF4-C/EBPβ heterodimer, other pro-atrophy transcription regulators also contribute to *Gadd45a* gene expression during skeletal muscle atrophy, including FOXO1, FOXO3, FOXO4, and HDAC4 (18, 21). Although many stress conditions induce *Gadd45a* expression in skeletal muscle, the degree of induction is context dependent; for example, the level of *Gadd45a* mRNA in skeletal muscle generally rises by 2- to 6-fold during relatively mild stress conditions such as immobilization, fasting, and aging (6, 11–13, 19–21) and by 20- to 60-fold during more severe stresses such as critical illness and muscle denervation (4, 21–23).

Induction of the *Gadd45a* gene increases expression of GADD45A, an 18 kDa globular protein (10). In mouse tibialis anterior (TA) muscle, forced expression of GADD45A is sufficient to induce muscle fiber atrophy (7). Conversely, RNAi-mediated knockdown of *Gadd45a* in mouse TA muscle reduces muscle fiber atrophy during several acute stress conditions, including fasting, limb immobilization, and ATF4 overexpression (7). In skeletal muscle fibers, GADD45A interacts with a group of signaling molecules that includes MEKK4, a member of the MAP kinase kinase kinase family (24). MEKK4 lacks protein kinase activity in the absence of GADD45A, but it undergoes a conformational change and becomes active upon GADD45A binding (24). Furthermore, MEKK4 kinase activity is essential for GADD45A-mediated muscle fiber atrophy (24).

Previous studies identified biochemical mechanisms upstream and downstream of GADD45A in skeletal muscle fibers, and they indicated that GADD45A has a capacity to induce muscle fiber atrophy in the TA muscle, a small muscle in the anterolateral lower leg. However, it remained unknown whether GADD45A had similar effects on other skeletal muscles and whether GADD45A is sufficient to generate functional deficits that are characteristic of muscle atrophy. In the current study, we sought to resolve these questions by generating and investigating transgenic mice with increased expression of GADD45A in a broad range of skeletal muscles.

## Results

### GADD45A expression in skeletal muscle fibers reduces muscle mass, strength, specific force, and exercise capacity.

**Figure 1:**
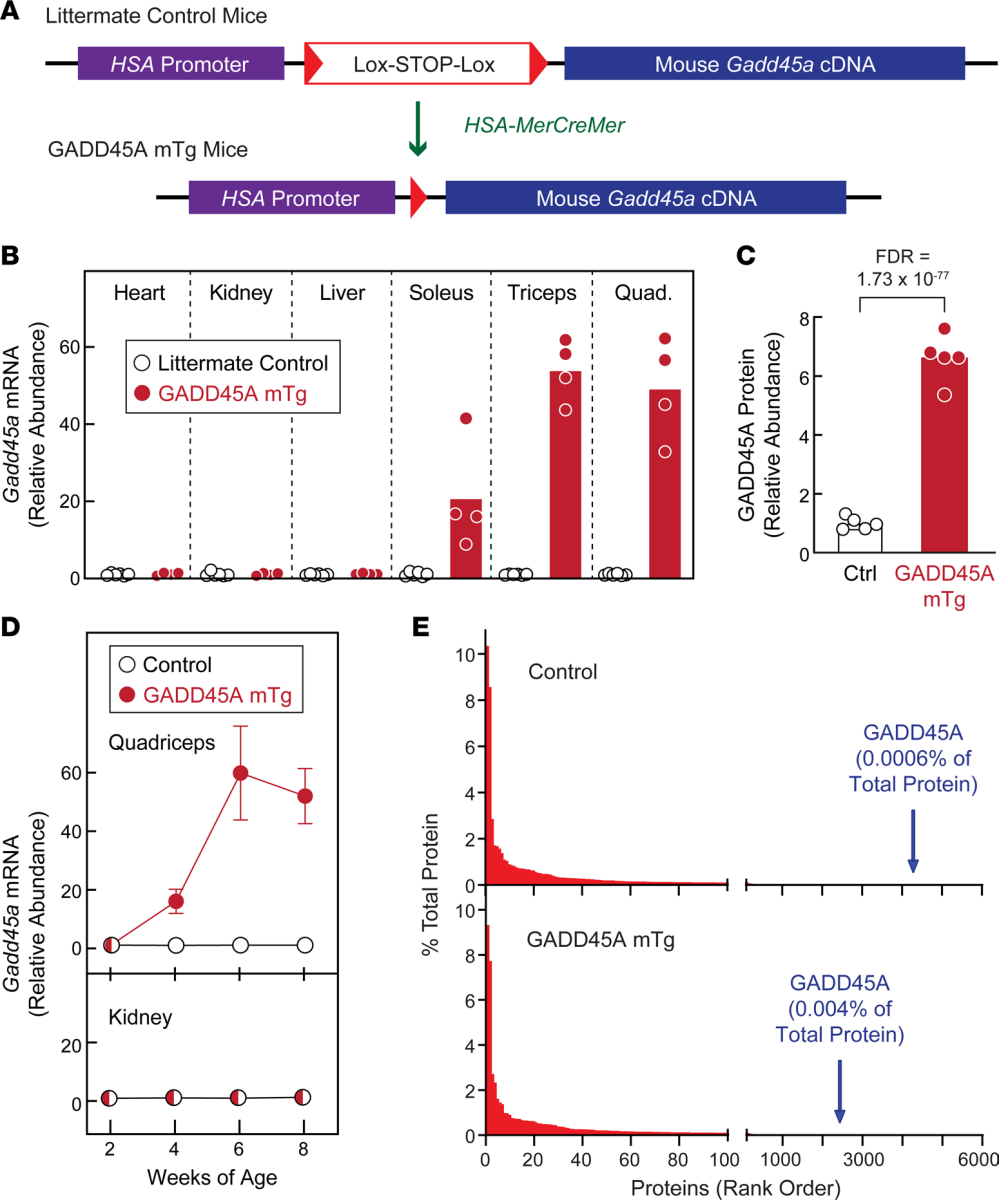
Transgenic mice with constitutive expression of GADD45A in skeletal muscle. (**A**) Schematic illustration of skeletal muscle–specific GADD45A-transgenic mice (GADD45A-mTg mice), which express a mouse *Gadd45a* cDNA under control of the skeletal muscle–specific (human *ACTA1* [*HSA*]) promoter. In GADD45A-mTg mice, a skeletal muscle–specific Cre recombinase transgene (*HSA-MerCreMer*) excises a *Lox-STOP-Lox* cassette upstream of the *Gadd45a* cDNA. In our studies of GADD45A-mTg mice, the control mice were littermates who lacked the *HSA-MerCreMer* transgene and thus retained the *Lox-STOP-Lox* cassette. (**B**) Heart, kidney, liver, soleus, triceps brachii (Triceps), and quadriceps femoris (Quad.) were collected from 15-month-old male littermate control and GADD45A-mTg mice, then subjected to quantitative PCR (qPCR) analysis of *Gadd45a* mRNA. Each circle represents the value from 1 animal, and bars indicate mean values. (**C**) Protein from quadriceps muscles of 15-month-old male littermate control (Ctrl) and GADD45A-mTg mice was subjected to tandem mass tag (TMT) labeling and mass spectrometry, followed by quantification of the relative abundance of GADD45A protein. Each circle represents the value from 1 animal, and bars indicate mean values. (**D**) Quadriceps and kidneys were harvested from littermate control and GADD45A-mTg mice at 2, 4, 6, and 8 weeks of age, then subjected to qPCR analysis of *Gadd45a* mRNA. Data are means ± SD from 2–3 control mice and 3–4 GADD45A-mTg mice per time point. Some error bars are too small to see. (**E**) Fractional abundance and rank order of all detected proteins, including GADD45A, in quadriceps muscle of 15-month-old male littermate control and GADD45A-mTg mice, as assessed by TMT-mass spectrometry.

To better understand the consequences of GADD45A expression in skeletal muscle fibers, we generated skeletal muscle–specific GADD45A-transgenic mice (GADD45A-mTg mice) (Figure 1A). We generated these mice by inserting a transgene containing a skeletal muscle fiber–specific promoter (human *ACTA1* [*HSA*] promoter), a *Lox-STOP-Lox* cassette, and mouse *Gadd45a* cDNA (Figure 1A) into the mouse *ROSA26* locus, then crossing homozygous *HSA-Lox-STOP-Lox-Gadd45a* mice to heterozygous *HSA-MerCreMer* mice, which express a tamoxifen-inducible Cre recombinase construct (MerCreMer) under control of the *HSA* promoter (25).

We employed *HSA-MerCreMer* mice based on their capacity to excise floxed alleles in an inducible and skeletal muscle–specific manner (25). Unexpectedly, we found that *HSA-MerCreMer* excised the *Lox-STOP-Lox* cassette in a tamoxifen-independent manner. Thus, in the absence of tamoxifen treatment, skeletal muscles of GADD45A-mTg mice contained an increased level of *Gadd45a* mRNA (Figure 1B) and 7-fold increased GADD45A protein expression (Figure 1C). Tamoxifen-independent *Gadd45a* mRNA expression was detectable by 4 weeks of age and plateaued by 6 weeks of age (Figure 1D). The degree to which *Gadd45a* mRNA was increased in GADD45A-mTg muscles was in the range observed in severe stress conditions, such as critical illness and muscle denervation, and higher than observed in more mild stress conditions, such as immobilization, fasting, and aging. Following tamoxifen-independent expression of the GADD45A transgene, GADD45A remained a minor component of the overall skeletal muscle proteome, representing 0.004% of total protein detected by mass spectrometry (Figure 1E).

Importantly, although excision of the *Lox-STOP-Lox* cassette was tamoxifen independent, it remained dependent on the presence of MerCreMer (Figure 1, B–E) and was skeletal muscle specific (Figure 1, B and D). Therefore, in all of our studies, GADD45A-mTg mice were compared with littermate control mice (which possessed the *HSA-Lox-STOP-Lox-Gadd45a* transgene but lacked the *HSA-MerCreMer* transgene), and no mice were treated with tamoxifen.

**Figure 2:**
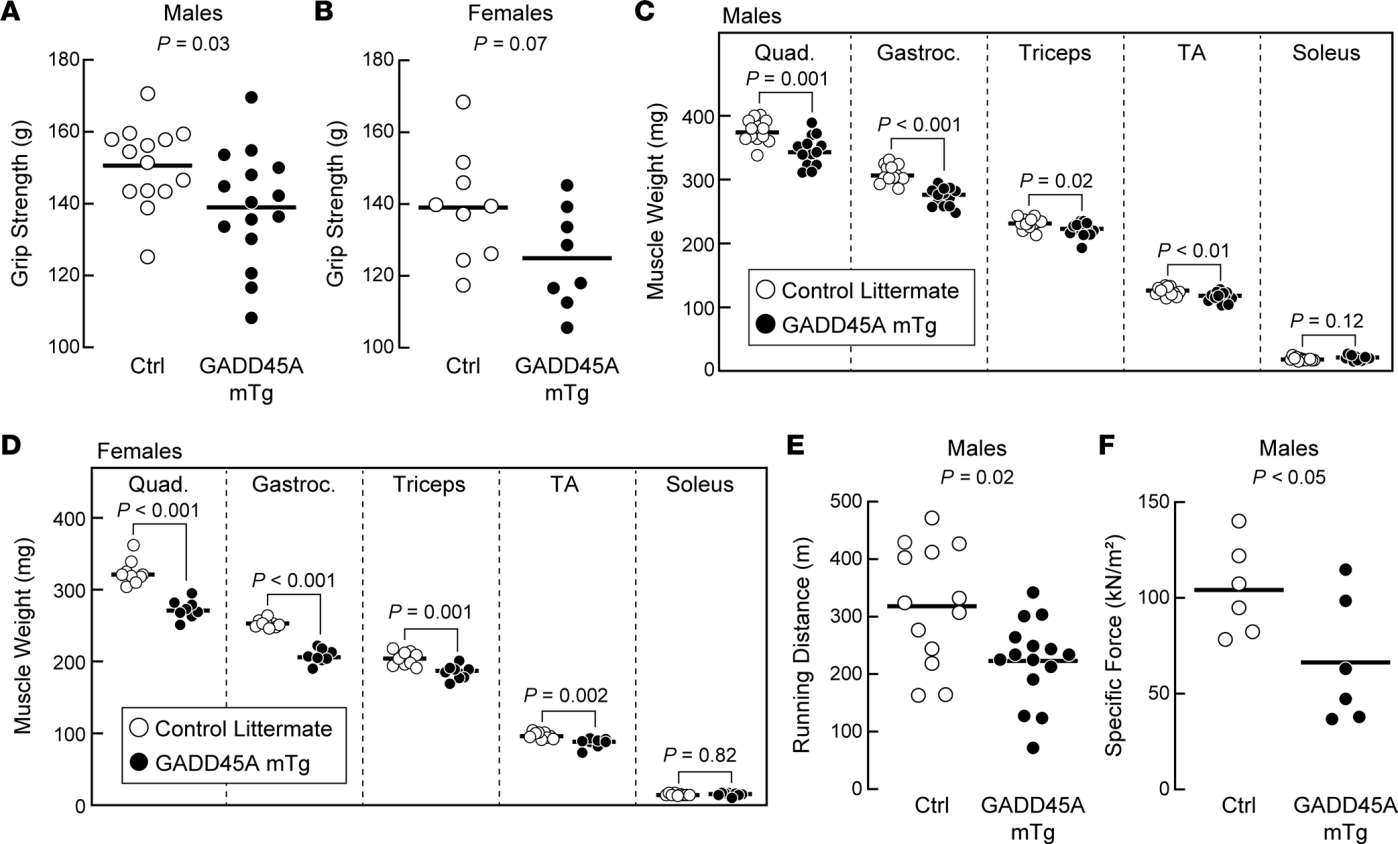
GADD45A expression in skeletal muscle reduces skeletal muscle mass and skeletal muscle function. (**A** and **B**) Grip strength in 15-month-old male (**A**) and 17-month-old female (**B**) littermate control and GADD45A-mTg mice. (**C** and **D**) Wet weights of bilateral quadriceps (Quad.), gastrocnemius (Gastroc.), triceps brachii (Triceps), tibialis anterior (TA), and soleus muscles in 15-month-old male (**C**) and 17-month-old female (**D**) littermate control and GADD45A-mTg mice. (**E**) Maximal treadmill running distance in 15-month-old male littermate control and GADD45A-mTg mice. (**F**) Ex vivo specific force in the extensor digitorum longus muscles of 15-month-old male littermate control and GADD45A-mTg mice. (**A**–**F**) Each circle represents the value from 1 animal, and horizontal bars indicate mean values. *P* values were determined with unpaired 2-tailed *t* tests.

Relative to control littermates, GADD45A-mTg mice exhibited no changes in survival, body weight, or weights of heart, liver, or fat pads to at least 17 months of age, the oldest age we investigated in this study (Supplemental Figure 1; supplemental material available online with this article; https://doi.org/10.1172/jci.insight.171772DS1; and not shown). However, in both female and male GADD45A-mTg mice, several skeletal muscle phenotypes were apparent. First, female and male GADD45A-mTg mice tended to be weaker than their control littermates (Figure 2, A and B). Second, female and male GADD45A-mTg mice had significantly smaller quadriceps femoris, triceps brachii, gastrocnemius, and TA muscles (Figure 2, C and D). We observed only 1 skeletal muscle whose mass was not reduced in GADD45A-mTg mice, the soleus (Figure 2, C and D). In addition, we investigated in vivo endurance exercise capacity and ex vivo specific force in male GADD45A-mTg mice, and both endurance exercise capacity and specific force were significantly reduced relative to control littermates (Figure 2, E and F). We observed similar phenotypes in younger, 8-month-old male GADD45A-mTg mice, including reduced grip strength; a trend toward reduced specific force (*P* = 0.06); reduced weights of TA, quadriceps, and gastrocnemius muscles; and increased weight of the soleus muscle (Supplemental Figure 2, A–F). Thus, increased GADD45A expression in skeletal muscle reduces the mass of some limb muscles (quadriceps, TA, gastrocnemius, triceps, and extensor digitorum longus, EDL) and impairs several aspects of muscle function, including strength, specific force, and endurance exercise capacity.

### GADD45A induces selective atrophy of large glycolytic skeletal muscle fibers and increases the size and relative amount of small skeletal muscle fibers that express MyHC-2A.

**Figure 3:**
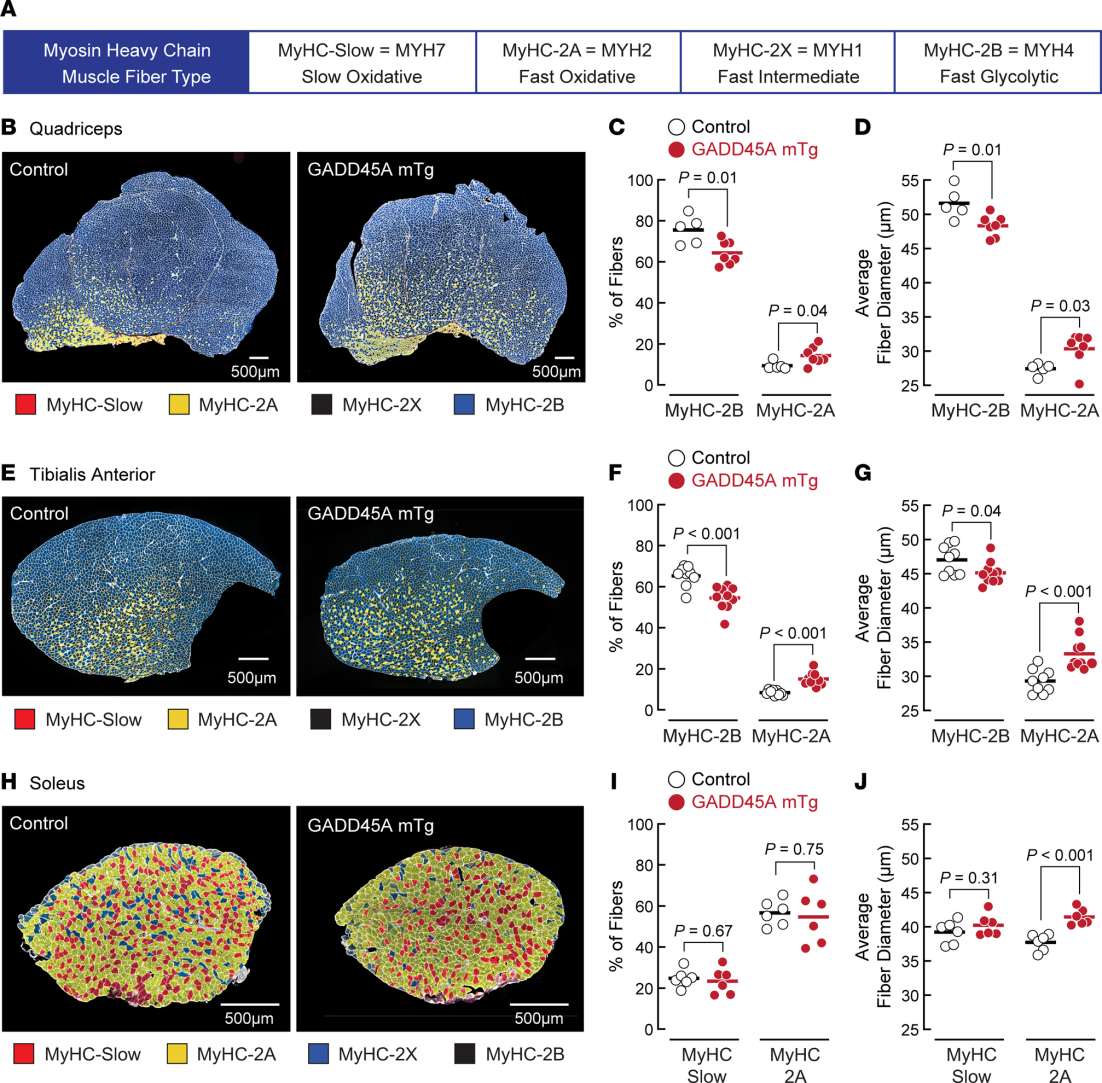
Chronic expression of GADD45A in skeletal muscle induces atrophy of large glycolytic MyHC-2B–positive muscle fibers and increases the size and relative amount of small MyHC-2A–positive muscle fibers. (**A**) MyHC nomenclature and typical characteristics of skeletal muscle fiber types that contain specific MyHC isoforms. (**B**–**G**) Quadriceps and TA muscles from 15-month-old male littermate control and GADD45A-mTg mice were sectioned and subjected to immunofluorescence microscopy using antibodies targeting laminin (white), MyHC-slow (red), MyHC-2A (yellow), and MyHC-2B (blue). Muscle fibers without MyHC-slow, MyHC-2A, and MyHC-2B were assigned to the unstained MyHC isoform (MyHC-2X). (**B**–**D**) Representative images (**B**), relative amounts of muscle fibers expressing MyHC-2B or MyHC-2A (**C**), and average minimal Feret diameters of muscle fibers expressing MyHC-2B or MyHC-2A (**D**) in the quadriceps. (**E**–**G**) Representative images (**E**), relative amounts of muscle fibers expressing MyHC-2B or MyHC-2A (**F**), and average minimal Feret diameters of muscle fibers expressing MyHC-2B or MyHC-2A (**G**) in the TA. (**H**–**J**) Soleus muscles from 15-month-old male littermate control and GADD45A-mTg mice were sectioned and subjected to immunofluorescence microscopy using antibodies targeting laminin (white), MyHC-slow (red), MyHC-2A (yellow), and MyHC-2X (blue). Muscle fibers without MyHC-slow, MyHC-2A, and MyHC-2X were assigned to the unstained MyHC isoform (MyHC-2B). Representative images (**H**), relative amounts of muscle fibers expressing MyHC-slow or MyHC-2A (**I**), and average minimal Feret diameters of muscle fibers expressing MyHC-slow or MyHC-2A (**J**). In **C**, **D**, **F**, **G**, **I**, and **J**, each data point represents the mean value from 1 muscle, horizontal bars indicate mean values from each group, and *P* values were determined with unpaired 2-tailed *t* tests.

To begin to investigate the mechanism by which GADD45A reduces skeletal muscle mass and function, we performed immunofluorescence microscopy on skeletal muscles from 15-month-old male GADD45A-mTg mice and male control littermates using antibodies directed against the 4 major myosin heavy chain (MyHC) isoforms in adult mouse muscle. In healthy mouse muscle, MyHC-slow (a.k.a. MYH7) is expressed in slow oxidative type I fibers, MyHC-2A (a.k.a. MYH2) is expressed in fast oxidative type IIA fibers, MyHC-2X (a.k.a. MYH1) is expressed in fast intermediate oxidative/glycolytic type IIx fibers, and MyHC-2B (a.k.a. MYH4) is expressed in fast glycolytic type IIB fibers (Figure 3A). In both control mice and GADD45A-mTg mice, the TA and quadriceps were mostly composed of large fibers that expressed MyHC-2B, and the remaining fibers were smaller and expressed either MyHC-2A or MyHC-2X (Figure 3, B, C, E, and F). In the soleus, most muscle fibers expressed either MyHC-2A or MyHC-slow, and MyHC-2B was not detectable (Figure 3, H and I).

Interestingly, in the TA and quadriceps, whose masses were reduced by GADD45A expression (Figure 2, C and D, and Supplemental Figure 2, C and D), GADD45A decreased the size and relative percentage of fibers that express MyHC-2B, while increasing the size and relative percentage of fibers that express MyHC-2A (Figure 3, B–G). We observed similar changes in TA muscles from 8-month-old male GADD45A-mTg mice (Supplemental Figure 2, G–I). In contrast, in the soleus, whose mass was unaffected by GADD45A expression (Figure 2, C and D, and Supplemental Figure 2F), GADD45A did not alter the relative percentage of MyHC-2A and MyHC-slow fibers. It did not alter the size of MyHC-slow fibers, but it significantly increased the size of MyHC-2A fibers (Figure 3, H–J). In all 3 of these muscles (TA, quadriceps, and soleus), GADD45A did not alter the total number of skeletal muscle fibers or elicit any consistent changes in the number or size of MyHC-2X fibers (not shown). Thus, GADD45A-mediated muscle atrophy in the TA and quadriceps was due to selective atrophy of large glycolytic fibers. Furthermore, GADD45A increased the size of small MyHC-2A–positive fibers in the TA, quadriceps, and soleus, and GADD45A increased the number of MyHC-2A–positive fibers in the TA and quadriceps.

**Figure 4:**
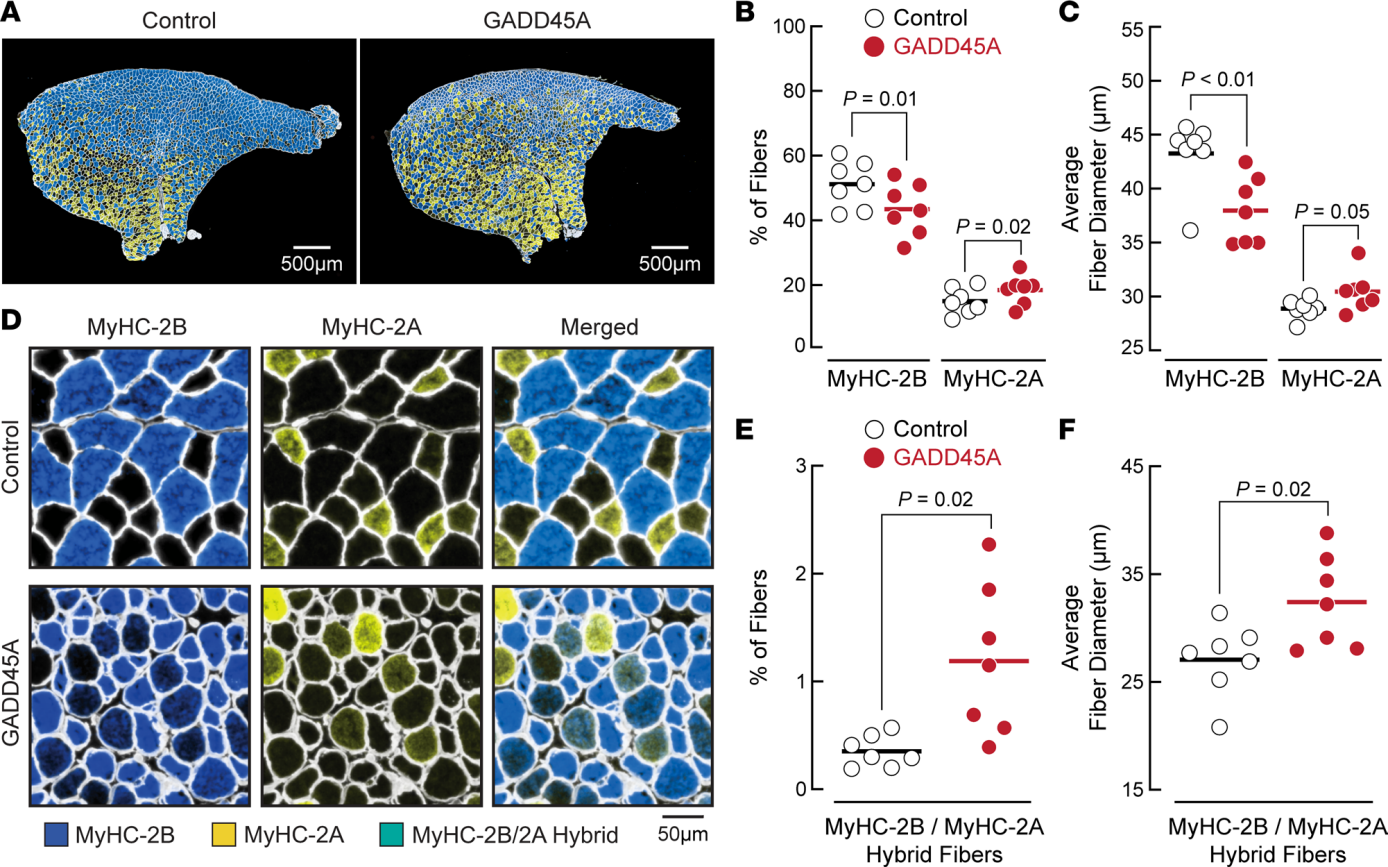
Acute expression of GADD45A in skeletal muscle induces atrophy of large, glycolytic muscle fibers and increases the size and relative amount of small MyHC-2A–positive muscle fibers. TA muscles of 8-week-old male C57BL/6 mice were transfected with plasmid DNA. One TA per mouse was transfected with 2.5 mg empty plasmid (control), and the contralateral TA in each mouse was transfected with 2.5 mg plasmid encoding mouse GADD45A. Seven days later, bilateral TAs were sectioned and subjected to immunofluorescence microscopy using antibodies targeting laminin, MyHC-2B, and MyHC-2A. (**A**) Representative images of entire TA muscle. MyHC-2B staining is blue, and MyHC-2A staining is yellow. (**B**) Relative amounts of muscle fibers expressing MyHC-2B or MyHC-2A. (**C**) Average minimal Feret diameters of muscle fibers expressing MyHC-2B or MyHC-2A. (**D**) Higher magnification images showing laminin staining (white), MyHC-2B staining (blue), MyHC-2A staining (yellow), and fibers expressing both MyHC-2B and MyHC-2A (green). (**E**) Relative amounts of muscle fibers expressing both MyHC-2B and MyHC-2A. (**F**) Average minimal Feret diameters of muscle fibers expressing both MyHC-2B and MyHC-2A. In **B**, **C**, **E**, and **F**, each data point represents the mean value from 1 muscle, horizontal bars indicate mean values from each group, and *P* values were determined with paired 2-tailed *t* tests.

The GADD45A mTg muscles used in this study had an elevated level of skeletal muscle GADD45A expression for several months, raising the possibility that the observed fiber type–specific changes might be the result of a slow process that requires chronic GADD45A expression. To test this possibility, we used an electroporation-based in vivo transfection method to introduce mouse *Gadd45a* cDNA into the TA muscles of 8-week-old C57BL/6 mice (6, 26) (Supplemental Figure 3). We then harvested the transfected skeletal muscles 1 week later for immunohistochemistry with MyHC-specific antibodies. Similar to TA muscles from GADD45A-mTg mice, 1 week of GADD45A expression in young adult TA muscles induced selective atrophy of large glycolytic fibers that express MyHC-2B, decreased the number of fibers that express MyHC-2B, and increased size and number of smaller fibers that express MyHC-2A (Figure 4, A–C). In this model, and in the GADD45A-mTg model, GADD45A induced some MyHC-2B fibers to become angular and severely atrophic, suggesting that GADD45A may contribute to a peripheral denervation process. Furthermore, in the presence of acute GADD45A expression, hybrid fibers expressing both MyHC-2B and MyHC-2A were apparent (Figure 4, D and E). These GADD45A -induced hybrid fibers were larger than MyHC-2B/2A hybrid fibers under control conditions (Figure 4F) and likely represent large glycolytic fibers transitioning to MyHC-2A expression. Additionally, in the presence of GADD45A, we observed evidence of hybrid fibers expressing both MyHC-2B and -2X, as well as hybrid fibers expressing both MyHC-2X and -2A (Supplemental Figure 4), suggesting a possibility that GADD45A may increase MyHC-2A through a classical sequence of MyHC transition from 2B to 2B/2X to 2X to 2X/2A to 2A (27). We observed similar findings when we acutely expressed the *Gadd45a* cDNA used in GADD45A-mTg mice, which contained N-terminal epitope tags (Supplemental Figure 5).

To determine if acute GADD45A expression might impair muscle function, we transfected *Gadd45a* cDNA into EDL muscles, which have a similar fiber type composition as the TA but are small enough to be amenable to ex vivo analyses of muscle force. We found that 1 week of *Gadd45a* plasmid expression in EDL muscles of 10-week-old adult C57BL/6 mice increased the level of *Gadd45a* mRNA by 6-fold and significantly decreased specific force, while decreasing the size of muscle fibers that express MyHC-2B and increasing the number of fibers that express MyHC-2A (Supplemental Figure 6). Collectively, these data indicate that an acute, short-term increase in GADD45A expression is sufficient to impair muscle function and induce the fiber type–specific changes observed in GADD45A-mTg muscles.

### GADD45A reduces oxidative capacity in glycolytic and oxidative skeletal muscle fibers.

In young, healthy skeletal muscle, MyHC-2A–positive skeletal muscle fibers (type IIA fibers) are rich in mitochondria and play an important role in oxidative metabolism and aerobic exercise capacity (27). Conversely, aging and acute stress conditions that cause muscle atrophy typically impair skeletal muscle mitochondria, oxidative metabolism, and aerobic exercise capacity (1, 2, 28–33). Based on these considerations, the finding that GADD45A increased the size and number of MyHC-2A–positive skeletal muscle fibers, while impairing skeletal muscle mass and function, seemed surprising and paradoxical.

**Figure 5:**
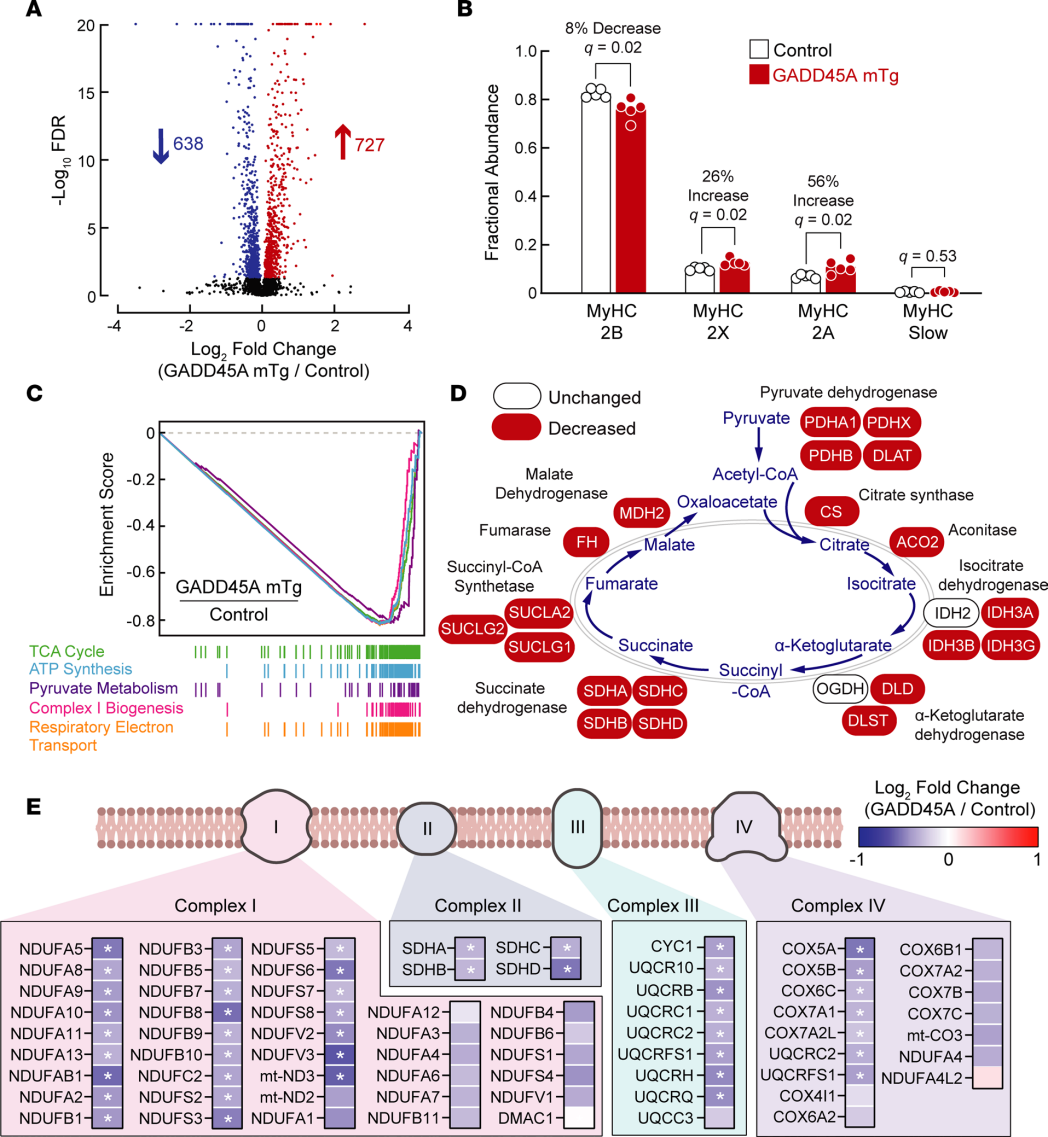
GADD45A reduces levels of numerous proteins that are required for oxidative metabolism in skeletal muscle. Protein from quadriceps muscles of 15-month-old male littermate control and GADD45A-mTg mice was subjected to TMT labeling and mass spectrometry, followed by quantification of the relative abundance of 5,206 proteins. Data are from 5 muscles per genotype. (**A**) Volcano plot showing log_2_ fold-changes of all identified proteins in GADD45A-mTg muscles relative to levels in control muscles versus statistical significance of those changes. At FDR < 0.05, levels of 638 proteins were decreased in GADD45A-mTg muscles, and levels of 727 proteins were increased. Complete proteomic data are shown in Supplemental Table 1. (**B**) Fractional abundance of MyHC-2B, MyHC-2X, MyHC-2A, and MyHC-slow, calculated from data in Supplemental Table 2. Each data point represents the value from 1 muscle, and bars indicate mean values. *q* values were determined with multiple unpaired 2-tailed *t* tests. (**C**) Enrichment plots of the 5 most significantly affected Reactome pathways, based on gene set enrichment analysis (GSEA) of the proteomic data. All 5 pathways were downregulated with FDR < 10^–5^. Complete GSEA results are shown in Supplemental Table 3. (**D**) Schematic illustrating detected proteins involved in the TCA cycle and effect of GADD45A on levels of those proteins. GADD45A significantly decreased 20 of the 22 detected proteins (FDR < 0.05). (**E**) Schematic of mitochondrial electron transport chain complexes I, II, III, and IV, with an underlying heatmap showing detected proteins in those complexes, and the effect of GADD45A on levels of those proteins. Asterisks indicate FDR < 0.05.

To further investigate this phenomenon, we used tandem mass tag TMT-mass spectrometry to quantify the levels of 5,206 proteins in the quadriceps of GADD45A-mTg and littermate control mice. Together, we identified 727 proteins whose levels were significantly increased by GADD45A expression and 638 proteins whose levels were significantly decreased by GADD45A expression (Figure 5A and Supplemental Table 1). As expected, and as discussed previously, GADD45A was one of the proteins whose level was increased in GADD45A-mTg quadriceps (Figure 1, C and E). Consistent with MyHC immunofluorescence in GADD45A-mTg quadriceps (Figure 3, B–D), we found that GADD45A-mTg quadriceps contained a decreased level of MyHC-2B and an increased level of MyHC-2A (Figure 5B and Supplemental Table 2). Additionally, by this proteomic analysis, we observed that GADD45A increased the level of MyHC-2X (Figure 5B and Supplemental Table 2), consistent with the notion that the transition from MyHC-2B to MyHC-2A expression may proceed through an intermediary step that involves MyHC-2X expression (27). Importantly, despite the increase in MyHC-2A expression, the levels of most proteins required for oxidative phosphorylation were strongly decreased. For example, pathway analysis of the proteomic data revealed that the 5 most strongly repressed cellular processes in GADD45A-mTg muscle were “TCA cycle,” “ATP synthesis,” “pyruvate metabolism,” complex I biogenesis,” and “respiratory electron transport chain” (Figure 5C and Supplemental Table 3). Consistent with this finding, GADD45A-mTg muscle contained significantly lower levels of 20 enzymes involved in the TCA cycle (Figure 5D), as well as significantly lower levels of 25 proteins from respiratory complex I, all 4 proteins from respiratory complex II/succinate dehydrogenase, 8 proteins from respiratory complex III, and 7 proteins from respiratory complex IV (Figure 5E). Together, we quantitated levels of 776 mitochondrial proteins contained in the mouse MitoCarta 3.0 database, and 244 of these proteins (31%) were significantly decreased in GADD45A-mTg muscles (FDR < 0.05; Supplemental Table 4).

**Figure 6:**
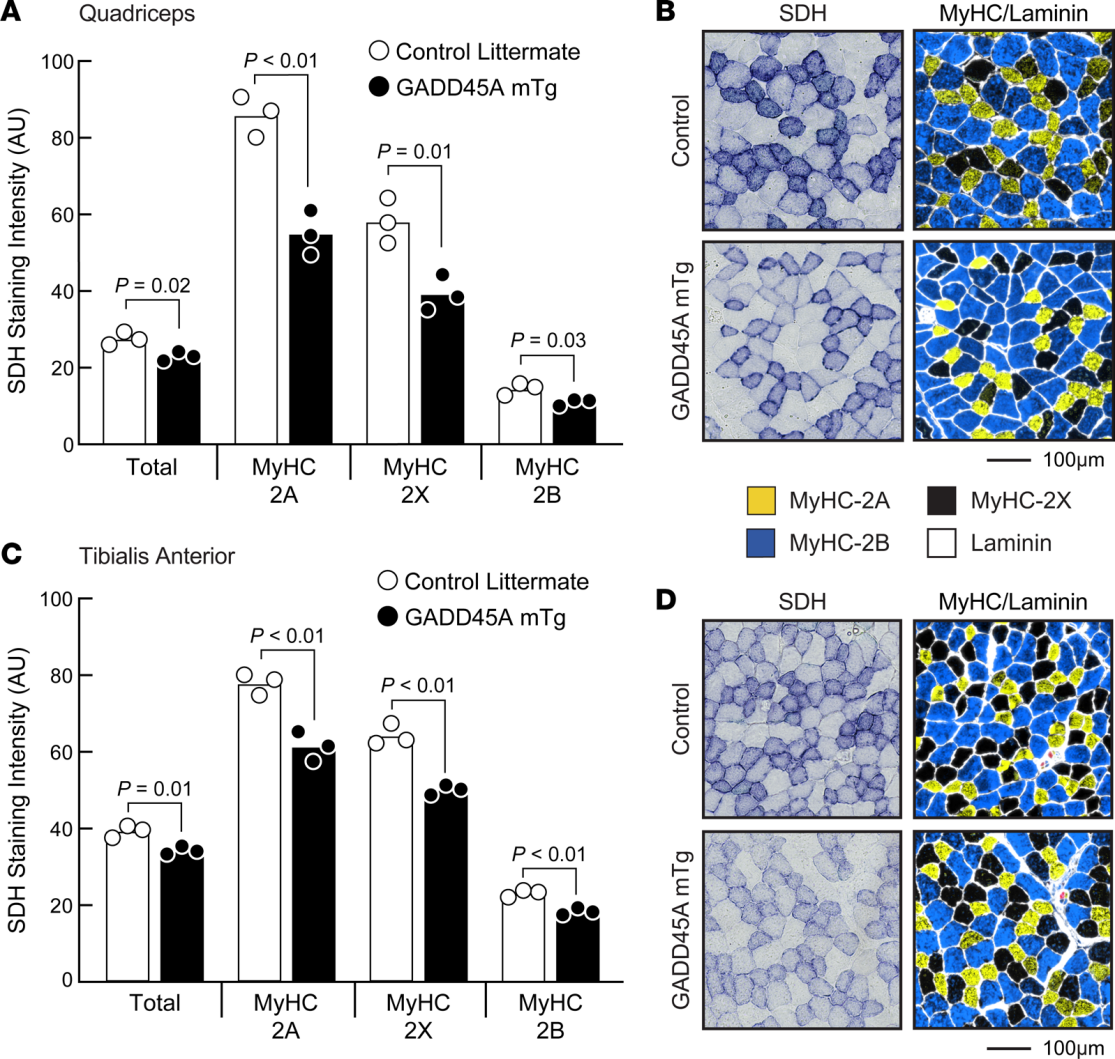
GADD45A reduces oxidative capacity in glycolytic and oxidative skeletal muscle fibers. Quadriceps and TA muscles from 15-month-old male littermate control and GADD45A-mTg mice were cryosectioned. Muscle cross sections were stained for succinate dehydrogenase (SDH) activity and then labeled with antibodies targeting MyHC isoforms and laminin. Cross sections were then imaged by bright-field and fluorescence microscopy to capture SDH activity and immunostaining, respectively. Captured images were then overlaid, and SDH activity and MyHC expression were quantitated in every fiber in the muscle, in order to determine SDH activity as a function of MyHC expression, as further illustrated in Supplemental Figure 7. (**A**) Quantification of MyHC expression versus SDH activity in quadriceps. (**B**) Representative SDH and MyHC/laminin stains of quadriceps cross sections from littermate control and GADD45A-mTg mice. (**C**) Quantification of MyHC and laminin expression versus SDH activity in TA. (**D**) Representative SDH and MyHC/laminin stains of TA cross sections from littermate control and GADD45A-mTg mice. In **A** and **C**, each data point represents the mean value from 1 muscle, bars indicate mean values from each group, and *P* values were determined with unpaired 2-tailed *t* tests.

To determine whether a reduction in oxidative capacity might be restricted to certain fiber types, we performed MyHC-based immunohistochemistry in conjunction with histochemical staining for succinate dehydrogenase (SDH) activity. As expected, in the quadriceps and TA muscles of control and GADD45A-mTg mice, SDH activity was highest in fibers that expressed MyHC-2A and lowest in fibers that expressed MyHC-2B, with an intermediate amount of SDH activity in fibers that expressed MyHC-2X (Figure 6, A–D, and Supplemental Figure 7). In all 3 of these fiber types, GADD45A reduced SDH activity, with the largest reduction occurring in MyHC-2A–positive fibers (Figure 6, A–D, and Supplemental Figure 7C). Similarly, in the soleus, GADD45A significantly reduced SDH activity in fibers that expressed MyHC-2A, MyHC-2X, and MyHC-slow (Supplemental Figure 8). Thus, GADD45A reduces oxidative capacity in all skeletal muscle fiber types.

**Figure 7:**
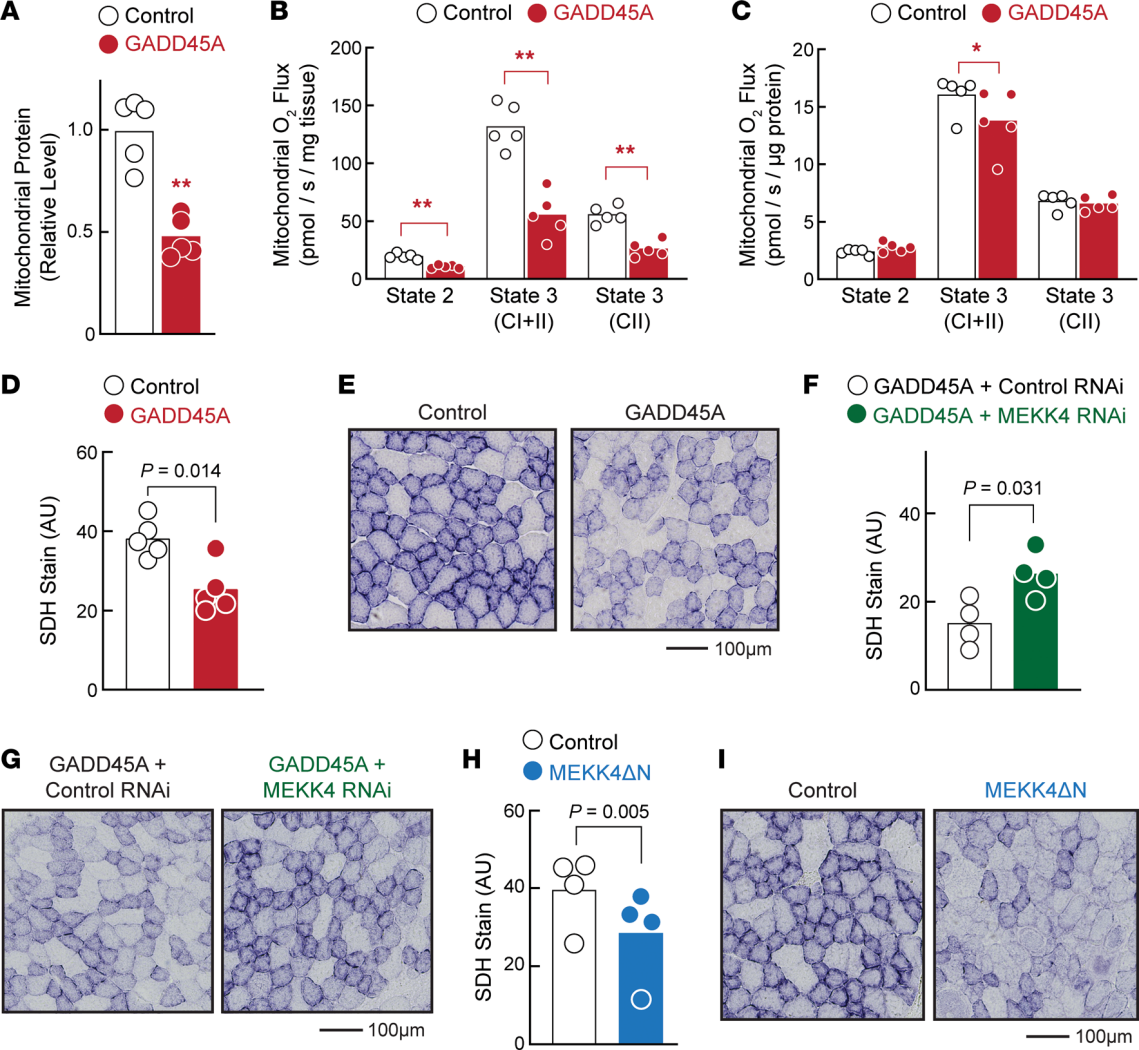
GADD45A reduces skeletal muscle oxidative capacity in an MEKK4-dependent manner. TA muscles of 8-week-old male C57BL/6 mice were transfected with plasmid DNA. (**A**–**E**) One TA per mouse was transfected with 2.5 mg empty plasmid (control), and the contralateral TA in each mouse was transfected with 2.5 mg GADD45A plasmid. (**A**–**C**) Seven days posttransfection, mitochondria from bilateral TAs were isolated and used for quantification of mitochondrial protein (**A**), mitochondrial respiration normalized to the amount of skeletal muscle (**B**), and mitochondrial respiration normalized to the amount of mitochondrial protein (**C**). (**D** and **E**) Seven days posttransfection, bilateral TAs were sectioned and subjected to immunohistochemical analysis of SDH activity. (**D**) Quantification of total SDH activity in entire muscle cross sections. (**E**) Representative images. (**F** and **G**) One TA per mouse was transfected with 2.5 mg GADD45A plasmid + 10 mg nontargeting control RNAi plasmid (control), and the contralateral TA in each mouse was transfected with 2.5 mg GADD45A plasmid + 10 mg RNAi plasmid targeting MEKK4. Seven days later, bilateral TAs were sectioned and subjected to immunohistochemical analysis of SDH activity. (**F**) Quantification of total SDH activity in entire muscle cross sections. (**G**) Representative images. (**H** and **I**) One TA per mouse was transfected with 7.5 mg empty plasmid (control), and the contralateral TA in each mouse was transfected with 7.5 mg plasmid encoding MEKK4ΔN. (**H** and **I**) Fourteen days posttransfection, bilateral TAs were sectioned and subjected to immunohistochemical analysis of SDH activity. (**H**) Quantification of total SDH activity in entire muscle cross sections. (**I**) Representative images. In **A**–**D**, **F**, and **H**, each data point represents the value from 1 muscle and bars indicate mean values. *P* values were determined with paired 2-tailed *t* tests. In **A**–**C**, **P < 0.01 and *P < 0.05.

To test the hypothesis that this reduction in oxidative capacity might occur over a short period, we transfected *Gadd45a* cDNA into TA muscles of 8-week-old C57BL/6 mice, then isolated mitochondria 1 week later to quantitate mitochondrial protein and assess maximal mitochondrial oxygen consumption by high-resolution respirometry. Consistent with findings from GADD45A-mTg muscles, we found that acute expression of GADD45A significantly reduced mitochondrial protein content (Figure 7A). In addition, GADD45A significantly reduced mitochondrial respiration when normalized to muscle weight (Figure 7B) and when normalized to the amount of mitochondrial protein (Figure 7C). Moreover, acute expression of GADD45A reduced histochemical staining for SDH activity (Figure 7, D and E), similar to the effect of chronic GADD45A expression in GADD45A-mTg muscles. Thus, GADD45A reduces the content and function of mitochondria in skeletal muscle, consistent with its phenotypic effects on skeletal muscle mass and function. Furthermore, these data indicate that GADD45A has a capacity to dissociate MyHC-2A expression from oxidative capacity, leading to a paradoxical increase in MyHC-2A expression despite a simultaneous decrease in mitochondrial content and activity.

In skeletal muscle fibers, GADD45A binds and activates the protein kinase MEKK4, and GADD45A-MEKK4 signaling is critical for GADD45A-mediated muscle fiber atrophy (24). We therefore hypothesized that MEKK4 might mediate the inhibitory effect of GADD45A on muscle oxidative capacity. As an initial test of this hypothesis, we used an RNAi construct that specifically reduces MEKK4 expression in mouse muscle fibers, leading to an inhibition of GADD45A-mediated muscle fiber atrophy (24). We coexpressed MEKK4 RNAi with GADD45A in mouse TA muscle fibers for 1 week, and in each animal, the contralateral TA muscle fibers coexpressed GADD45A and a nontargeting control RNAi construct. In muscle fibers expressing GADD45A and the nontargeting control RNAi construct, SDH activity was low, as expected (Figure 7, F and G). However, MEKK4 RNAi inhibited this effect of GADD45A, leading to preservation of SDH activity (Figure 7, F and G). To determine if MEKK4 activity might be sufficient to decrease oxidative capacity, we investigated the effect of MEKK4ΔN, a constitutively active MEKK4 construct that induces skeletal muscle fiber atrophy in a GADD45A-independent manner (24). Similar to GADD45A, MEKK4ΔN reduced SDH activity in skeletal muscle fibers (Figure 7, H and I). These data indicate that GADD45A reduces skeletal muscle oxidative capacity by activating MEKK4.

**Figure 8:**
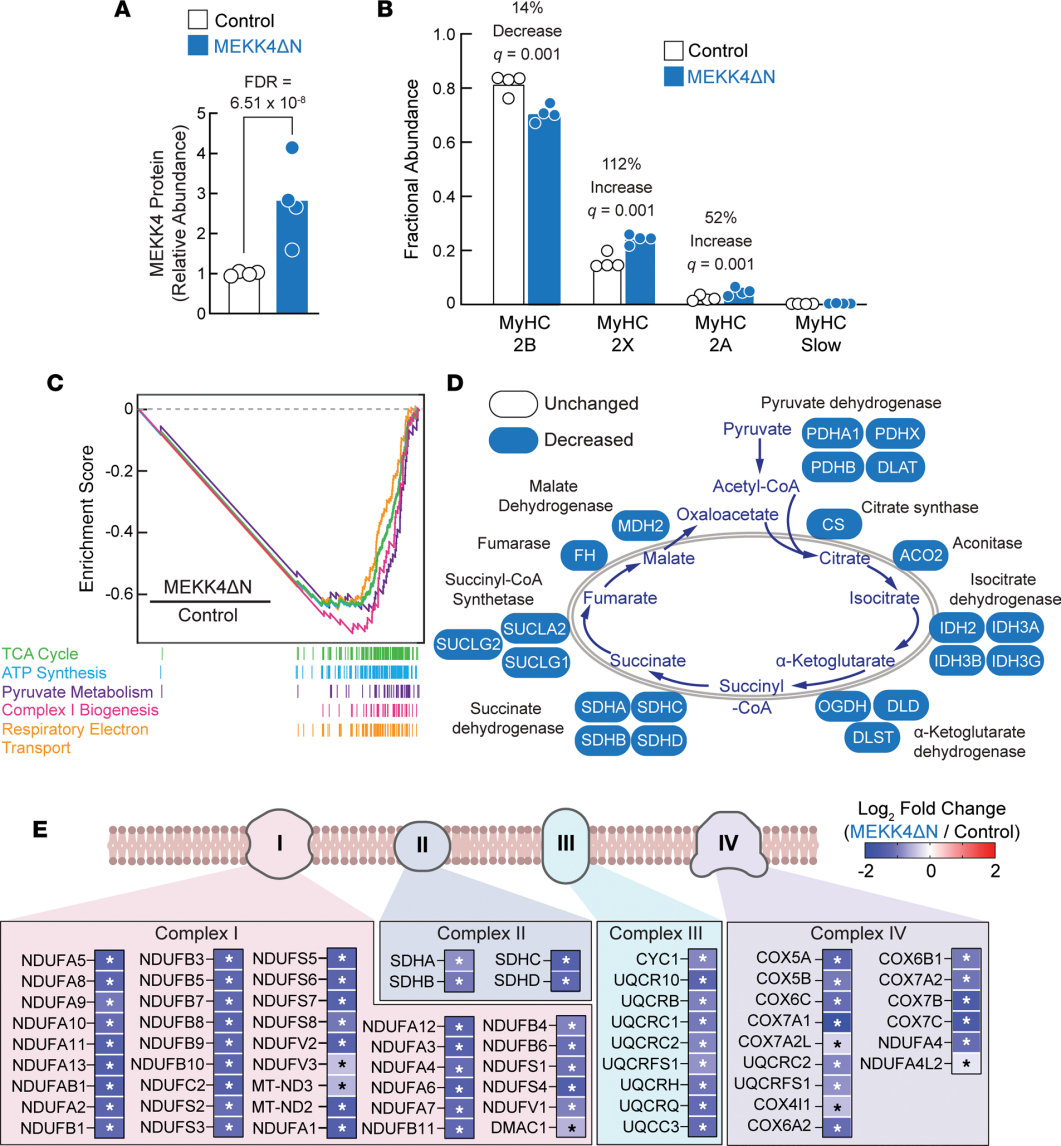
A constitutively active MEKK4 construct (MEKK4ΔN) decreases MyHC-2B, increases MyHC-2A, and decreases mitochondrial proteins in skeletal muscle. TA muscles of 8-week-old male C57BL/6 mice were transfected with plasmid DNA. One TA per mouse was transfected with 7.5 mg empty plasmid (control), and the contralateral TA in each mouse was transfected with 7.5 mg plasmid encoding MEKK4ΔN. Seven days posttransfection, bilateral TAs were harvested, and TA protein was subjected to TMT-mass spectrometry, followed by quantification of the relative abundance of 6,870 proteins. Data are from 4 muscles per genotype. (**A**) Quantification of MEKK4 (MAP3K4) protein. Each data point represents the value from 1 muscle, and bars indicate mean values. Detailed proteomic data are shown in Supplemental Table 5. (**B**) Fractional abundance of MyHC-2B, MyHC-2X, MyHC-2A, and MyHC-slow, calculated from data in Supplemental Table 6. Each data point represents the value from 1 muscle, and bars indicate mean values. *q* values were determined with multiple unpaired 2-tailed *t* tests. (**C**) Enrichment plots of Reactome pathways that were strongly repressed by MEKK4ΔN, based on GSEA of the proteomic data. All 5 pathways were downregulated with FDR < 10^–5^. Complete GSEA results are shown in Supplemental Table 7. (**D**) Schematic illustrating detected proteins involved in the TCA cycle and effect of MEKK4ΔN on levels of those proteins. MEKK4ΔN significantly decreased all the detected proteins (FDR < 0.05). (**E**) Schematic of mitochondrial electron transport chain complexes I, II, III, and IV, with an underlying heatmap showing detected proteins in those complexes, and the effect of MEKK4ΔN on levels of those proteins. Asterisks indicate FDR < 0.05.

To test the hypothesis that MEKK4 activity may decrease mitochondrial proteins, we expressed MEKK4ΔN in TA muscles of 8-week-old C57BL/6 mice for 1 week, then used TMT-mass spectrometry to quantify levels of skeletal muscle proteins. Interestingly, MEKK4ΔN expression (Figure 8A and Supplemental Table 5) significantly decreased MyHC-2B and increased MyHC-2A and MyHC-2X (Figure 8B and Supplemental Table 6), similar to GADD45A (Figure 5B). In addition, MEKK4ΔN strongly repressed mitochondrial pathways and significantly decreased levels of major mitochondrial proteins (Figure 8, C–E, and Supplemental Table 7), also similar to GADD45A (Figure 5, C–E). Thus, MEKK4 activity can account for important effects of GADD45A, including a reduction in MyHC-2B, an increase in MyHC-2A, and strong repression of mitochondrial proteins.

### Skeletal muscle GADD45A expression is associated with muscle weakness in humans.

**Figure 9:**
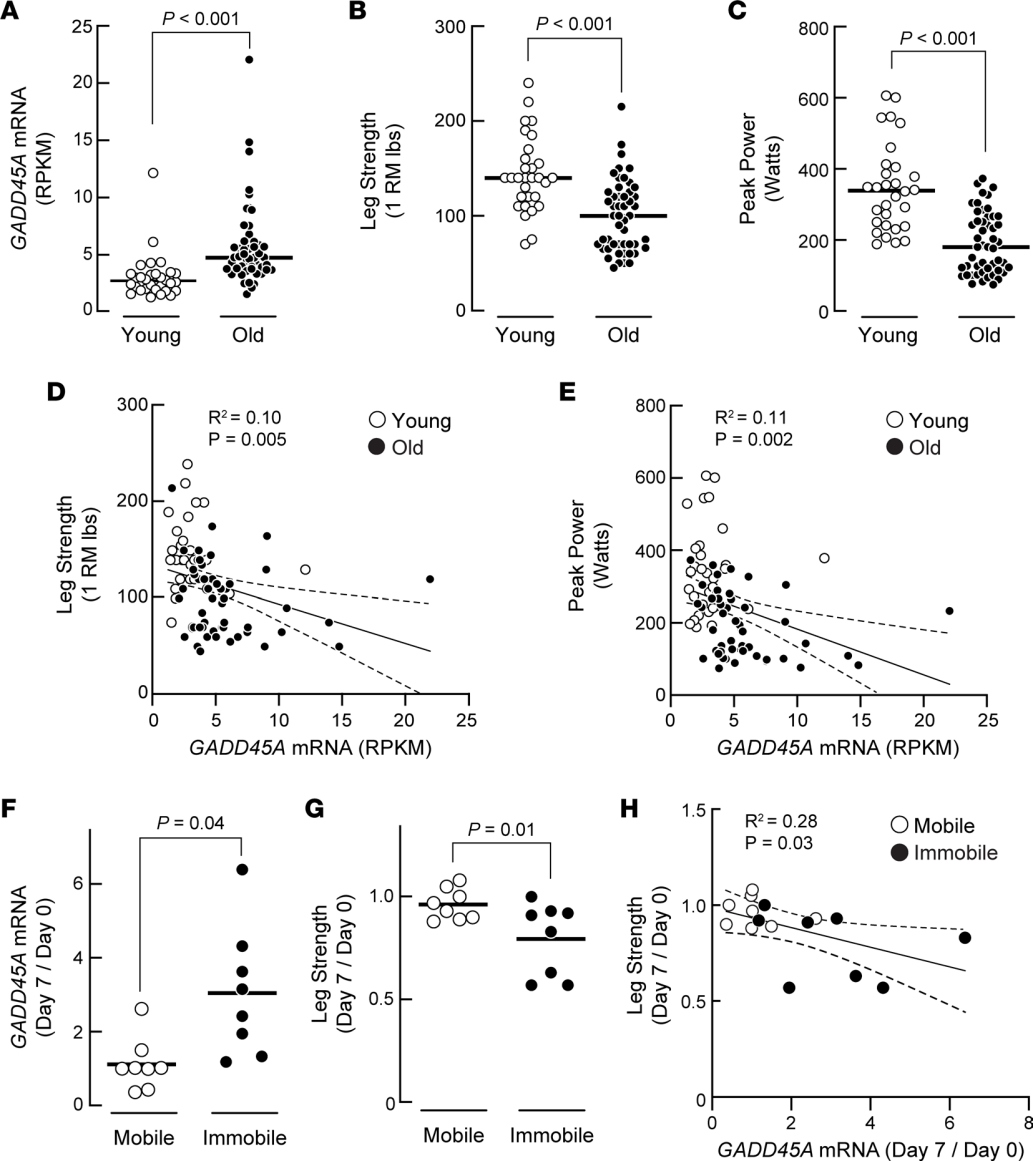
Skeletal muscle *GADD45A* expression is associated with muscle weakness in humans. (**A**–**E**) Human participants who were 20 to 35 years old (young) or 65 to 85 years old (old) volunteered for a study in which *GADD45A* mRNA levels in the vastus lateralis muscle were assessed by RNA sequencing and muscle function was assessed by measurement of maximal knee extensor strength and peak power. Each data point represents the value from 1 participant. See Supporting Data Values file. (**A**) *GADD45A* mRNA levels. (**B**) Maximal leg strength, assessed via 1 repetition maximum (1 RM) leg extension, a dynamic movement through a full range of motion of knee extension. (**C**) Peak leg power production. (**A**–**C**) Horizontal bars indicate mean values, and *P* values were determined with unpaired 2-tailed *t* tests. (**D** and **E**) Correlation of *GADD45A* mRNA levels to maximal leg strength (**D**) and peak leg power (**E**). (**F**–**H**) Human participants aged 50 to 63 years old volunteered for a study using a model of disuse muscle atrophy. In each participant, one leg was immobilized with a leg brace for 7 days, and the contralateral leg remained mobile and served as an intra-individual control. In both legs, maximal leg strength and *GADD45A* mRNA in the vastus lateralis muscle were quantified at baseline (day 0, prior to unilateral leg immobilization) and again on day 7 (after unilateral leg immobilization). Each data point represents the fold change (day 7/day 0) from the mobile or immobile leg, as indicated. (**F** and **G**) Fold-change in *GADD45A* mRNA levels (**F**) and maximal leg strength (**G**). Horizontal bars indicate mean values. *P* values were determined with paired 2-tailed *t* tests. (**H**) Correlation of *GADD45A* mRNA to maximal leg strength. (**D**, **E**, and **H**) Pearson’s correlation coefficient and *P* value were determined with simple linear regression. RPKM, reads per kilobase million.

We hypothesized that *GADD45A* expression may be associated with weakness in humans. As an initial test of this hypothesis, we quantified maximal leg strength, peak leg power, and *GADD45A* mRNA levels in the quadriceps muscle (vastus lateralis) in women and men who were either 20 to 35 years old (30 individuals) or 65 to 85 years old (50 individuals). We found that *GADD45A* mRNA was significantly increased in older human muscle (Figure 9A), consistent with previous reports (11, 12). Importantly, the level of *GADD45A* mRNA was negatively associated with maximal leg strength and peak leg power during dynamic unilateral knee extension (Figure 9, B–E). We observed similar findings with a smaller independent cohort of 12 young and 11 older individuals, including increased *GADD45A* mRNA in older human muscle and a significant negative correlation of *GADD45A* mRNA to leg strength (Supplemental Figure 9). In addition, in that smaller cohort, we assessed mitochondrial function in skeletal muscle, and the level of *GADD45A* mRNA tended to be associated with a reduction in state 3 mitochondrial respiration in the vastus lateralis muscles of these individuals (*P* = 0.11; Supplemental Figure 9). As an additional test of the hypothesis that *GADD45A* expression is associated with weakness in humans, we studied 5 women and 3 men (ages 50 to 63 years old) in a model of disuse muscle atrophy. In each participant, one leg was immobilized with a leg brace for 7 days, and the contralateral leg remained mobile and served as an intra-individual control. We quantified leg strength and *GADD45A* mRNA in the quadriceps muscle in both legs at baseline (day 0, prior to unilateral leg immobilization) and again on day 7 (after unilateral leg immobilization). We found that 1 week of immobilization significantly increased *GADD45A* mRNA (Figure 9F) and significantly decreased leg strength (Figure 9G). Furthermore, the induction of *GADD45A* mRNA was significantly associated with a reduction in leg strength (Figure 9H). These data, coupled with data from mouse models, suggest GADD45A as a potential mediator of weakness in human skeletal muscle.

## Discussion

**Figure 10:**
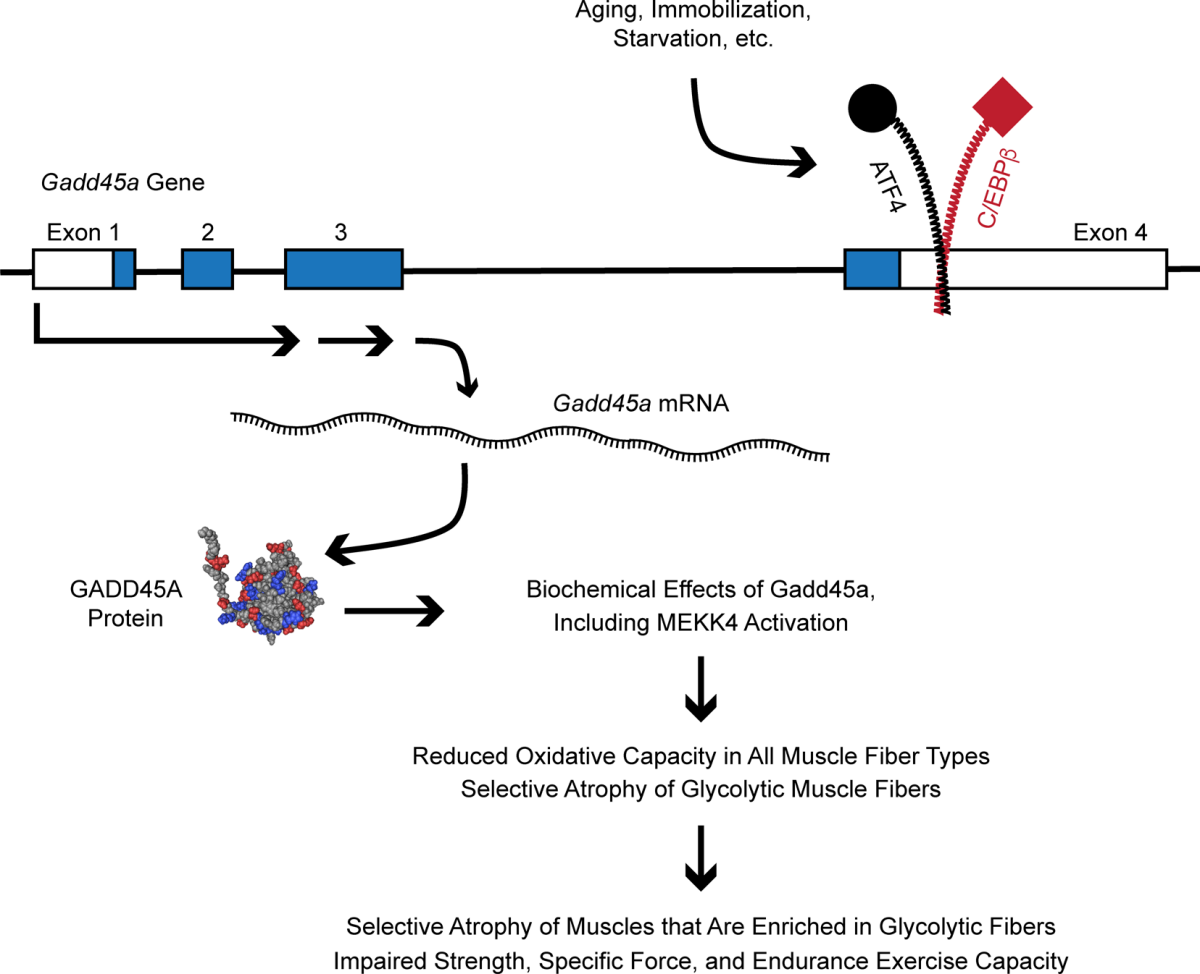
Schematic illustrating biochemical mechanisms upstream and downstream of GADD45A in skeletal muscle fibers and their cellular and phenotypic effects. The NMR structure of GADD45A was identified in ref. 45.

In the current study, we sought to better understand the role of GADD45A in skeletal muscle atrophy by investigating cellular and phenotypic consequences of GADD45A overexpression in skeletal muscle fibers. The results of this study, coupled with previous findings, suggest a model that is illustrated in Figure 10. In healthy skeletal muscle, the *Gadd45a* gene is relatively inactive. However, during conditions that cause muscle atrophy, the ATF4-C/EBPβ heterodimer and other pro-atrophy transcription factors activate the *Gadd45a* gene within skeletal muscle fibers (7, 10, 18, 21). This increases the level of GADD45A, a small globular protein that allosterically activates MEKK4, generating an active MAP kinase kinase kinase complex composed of both MEKK4 and GADD45A (24). Through mechanisms that at least partly involve MEKK4, GADD45A reduces oxidative capacity in all muscle fiber types and induces selective atrophy of glycolytic muscle fibers. By inducing these cellular changes, GADD45A decreases the mass of skeletal muscles that are enriched in glycolytic fibers, and it impairs important aspects of skeletal muscle function, including strength, specific force, and endurance exercise capacity. Furthermore, as predicted by data from mouse models, skeletal muscle *GADD45A* expression is associated with muscle weakness in humans. These findings suggest GADD45A as a mediator of skeletal muscle atrophy and weakness in mice and humans.

A reduction in skeletal muscle oxidative capacity is considered a central cellular mechanism of muscle atrophy and weakness (1, 2, 28–33). The current data indicate that GADD45A/MEKK4 signaling reduces muscle oxidative capacity, and it does so by decreasing levels of numerous proteins that are critical for mitochondrial function. The mechanism by which GADD45A/MEKK4 signaling decreases levels of mitochondrial proteins is not yet known. In muscle fibers, the GADD45A/MEKK4 complex activates 4 downstream MAP kinase kinases (MKK3, MKK4, MKK6, and MKK7), leading to activation of p38 MAP kinase (24). However, because MKK/p38 signaling promotes mitochondrial biogenesis in skeletal muscle (34, 35), it seems likely that other GADD45A/MEKK4 targets are involved. Indeed, unbiased protein purification studies have identified several proteins that directly and specifically interact with GADD45A in mouse muscle fibers in vivo, including not just MEKK4 but also 10 other protein kinases (RAF-1, A-RAF, JAK1, ILK, RSK2, SPEG, MSK1, MSK2, RIOK3, and RIPK3) and 3 protein tyrosine phosphatases (ACP1, PTP-1B, and SHP-1) (24). Additional GADD45A and MEKK4 targets may also remain to be discovered. In future investigations, it will be important to obtain a more detailed understanding of biochemical mechanisms downstream of GADD45A and how those mechanisms reduce synthesis and/or increase degradation of mitochondrial proteins in skeletal muscle.

In addition to its effect on mitochondrial proteins and oxidative capacity, GADD45A generates a second cellular change that is often observed in muscle atrophy: selective atrophy of fast glycolytic skeletal muscle fibers (1, 36–41). Atrophy of fast glycolytic fibers explains how GADD45A decreases the mass of muscles that are enriched in fast glycolytic fibers (quadriceps, TA, gastrocnemius, and triceps) but does not induce atrophy of the soleus muscle, which is largely devoid of fast glycolytic fibers. It also raises the question of how selective muscle fiber atrophy can occur. One possibility is that fast glycolytic fibers are more sensitive to impairments in mitochondrial function, given their lower baseline content of mitochondria. Another possibility is that glycolytic fibers may uniquely possess other factors that are essential for GADD45A-mediated muscle fiber atrophy, such as MEKK4, certain GADD45A/MEKK4 targets (MKK3, MKK4, MKK6, MKK7, or specific p38 isoforms), other known GADD45A-interacting proteins (e.g., RAF-1, A-RAF, JAK1, ILK, RSK2, SPEG, MSK1, MSK2, RIOK3, RIPK3, ACP1, PTP-1B, SHP-1, etc.), or other pro-atrophy signaling pathways that have not yet been linked to GADD45A signaling. As one example, NF-κB signaling in skeletal muscle fibers generates a similar pattern of atrophy as GADD45A signaling, with selective atrophy of glycolytic muscles and preservation of the soleus (42). Although the downstream biochemical mechanisms are not yet well understood, the current data identify GADD45A as a molecular mediator of both mitochondrial dysfunction and selective atrophy of glycolytic muscle fibers, 2 important cellular mechanisms of skeletal muscle atrophy and weakness.

Interestingly, GADD45A also induces a third cellular change that is often observed in skeletal muscle atrophy: a striking dissociation between actual oxidative capacity and expression of MyHC isoforms that, in healthy skeletal muscle, are characteristic of oxidative muscle fibers (1, 27, 28, 36, 43). This is most clearly observed with MyHC-2A expression, which is restricted to small, oxidative type IIA muscle fibers in healthy muscle. By decreasing levels of numerous proteins that are critical for mitochondrial function, GADD45A decreases oxidative capacity in most if not all muscle fibers. GADD45A also induces selective atrophy of glycolytic muscle fibers, a process that involves increased turnover of MyHC-2B. However, as GADD45A induces atrophy of glycolytic fibers, some of those fibers begin to express MyHC-2A, despite reduced oxidative capacity. Thus, GADD45A has a capacity to dissociate mitochondrial function from oxidative MyHC expression, a paradoxical phenomenon that is often observed in natural forms of skeletal muscle atrophy that are associated with increased GADD45A expression. How GADD45A generates a disconnect between mitochondrial function and oxidative myosin heavy chain expression is not yet known but may involve calcineurin, which is known to stimulate MyHC-2A expression (27) and was significantly increased in the quadriceps of GADD45A mTg muscles (Supplemental Table 1). The use of whole quadriceps muscles in our proteomic analyses precludes us from determining whether calcineurin and MyHC-2A increased within the same cells. Additional studies will be required to understand the downstream mechanism of MyHC-2A expression in atrophying glycolytic muscle fibers, but the current study identifies GADD45A as a molecular mediator of this effect.

By stimulating MyHC-2A expression in glycolytic fibers that are undergoing atrophy, GADD45A increases the number of fibers that express MyHC-2A and decreases the number of fibers that express MyHC-2B. In addition, GADD45A unexpectedly increases the size of fibers that express MyHC-2A. Larger MyHC-2A fibers in the quadriceps and TA can perhaps be partly explained by induction of MyHC-2A expression in glycolytic fibers that are atrophic but still larger than bona fide type IIA fibers. However, this explanation alone is insufficient, because GADD45A also increases the size of MyHC-2A fibers in the soleus, which suggests hypertrophy of bona fide type IIA fibers. Importantly, because type IIA fibers are small, comprise a relatively small portion of large muscles, and only slightly increase in size in the presence of GADD45A, the effect of GADD45A on type IIA fiber size has minimal effect on muscle mass. Nevertheless, this phenomenon is interesting and illustrates the cellular complexity of muscle atrophy, the limitations of traditional fiber typing methods, especially during muscle atrophy, and the lack of correlation between changes in oxidative capacity (which is decreased by GADD45A in fibers that express MyHC-2B, MyHC-2A, MyHC-2X, or MyHC-slow) and changes in muscle fiber size (which is decreased by GADD45A in fibers that express MyHC-2B, increased by GADD45A in fibers that express MyHC-2A, and unaffected by GADD45A in fibers that express MyHC-2X or MyHC-slow).

The cellular changes induced by GADD45A ultimately lead to several phenotypes, including atrophy of skeletal muscles that are enriched in glycolytic fibers and impairments in strength, specific force, and endurance exercise capacity. However, it is also important to emphasize that the phenotypes observed in GADD45A-mTg mice are mild relative to natural skeletal muscle atrophy in its most advanced and severe stage, even though GADD45A-mTg muscles contain a level of *Gadd45a* expression comparable to the most severe natural forms of muscle atrophy. This finding reflects the molecular complexity of skeletal muscle atrophy, which involves a multitude of molecular mediators and signaling pathways, including not just GADD45A but also other ATF4-C/EBPβ gene targets and many other mediators and pathways that operate in parallel to the ATF4-C/EBPβ/GADD45A pathway (1–4). A long-term challenge for the field is to reach an integrated and comprehensive understanding of the molecular mechanisms of muscle atrophy, including how the various molecular mediators and signaling pathways interact to generate all the cellular and phenotypic features of skeletal muscle atrophy. That larger understanding will require, among other things, an understanding of how each molecular mediator can contribute to muscle atrophy and weakness in various conditions that cause muscle atrophy. At a high level, the current data strongly suggest that induction of *Gadd45a* gene expression in muscle fibers could contribute to the overall process of muscle atrophy and weakness during a wide range of conditions (i.e., conditions associated with increased GADD45A expression), but GADD45A cannot by itself explain the entire disease process, consistent with the well-established existence of GADD45A-independent mechanisms, which also, like GADD45A, play contributory roles.

We believe the results of this study are interesting and important, but the study also has some limitations. First, as discussed above, the biochemical mechanisms downstream of GADD45A are incompletely understood, particularly across various muscle fiber types. Second, we intended that our GADD45A-mTg mice would be amenable to inducible control of GADD45A expression, but they were not, due to unexpected tamoxifen-independent activity of the *HSA-MCM* transgene. Although this limitation of the GADD45A-mTg model was partly overcome by complementary experiments that introduced GADD45A into TA muscle fibers of young adult C57BL/6 mice, the muscle transfection method is not 100% efficient and likely underestimates the full impact of GADD45A. Thus, additional efforts to generate inducible, skeletal muscle–specific GADD45A-transgenic mouse models would be warranted in the future, in order to better understand the phenotypic consequences of an acute increase in GADD45A expression in multiple muscle types and at multiple ages. In addition, because the level of *Gadd45a* mRNA in the GADD45A mTg model is in the range observed in conditions such as critical illness and muscle denervation, but much higher than observed in more mild stress conditions, such as immobilization, fasting, and aging, it would be helpful in the future to have models where GADD45A expression can be finely controlled and adjusted to match the levels of GADD45A expression that occur across natural atrophy conditions.

A third limitation of the current study is that, although it begins to extend the work from mouse models to humans, more comprehensive human investigations will be needed in the future to better understand the relationship of skeletal muscle GADD45A expression to clinically important outcomes during aging and other prevalent causes of muscle atrophy, such as critical illness, uncontrolled diabetes, cancer, renal failure, heart failure, chronic pulmonary disease, weight loss medications and surgeries, Cushing syndrome, chronic infections such as HIV/AIDS, osteoarthritis and other orthopedic conditions, rheumatoid arthritis and other systemic autoimmune conditions, and spinal cord injury. It is also important to recognize that each assessment of muscle function has certain limitations. For example, voluntary assessments of strength in humans or mice reflect not just the contractile properties of muscle but also other factors, including but not limited to motor unit recruitment pattern, muscle fiber architecture, fiber type distributions, peripheral excitation, and completeness of activation by the central nervous system. As another example, ex vivo assessments of specific force rely upon certain assumptions, including specific relationships between muscle length and fiber length, and between muscle cross-sectional area and muscle weight, that may not be entirely true during muscle atrophy. Thus, to best understand the relationship of skeletal muscle GADD45A expression to clinically meaningful outcomes, it will continue to be important to use a variety of complementary assays in both humans and mouse models.

Another limitation of the current study is that it demonstrates that GADD45A expression in muscle fibers is sufficient to induce several cellular and phenotypic changes that are characteristic of muscle atrophy, but it does not determine whether GADD45A is necessary for these effects. Previous studies have shown that acute RNAi-mediated knockdown of GADD45A reduces muscle fiber atrophy in the TA during fasting, limb immobilization, muscle denervation, and ATF4 overexpression (7). And yet, studies have also shown that gastrocnemius and soleus muscles of Gadd45a-knockout mice are not resistant to denervation-induced skeletal muscle atrophy (23). The previous finding that soleus muscles of Gadd45a-knockout mice are not resistant to denervation-induced atrophy can be explained by the current data that GADD45A does not induce atrophy in the soleus. However, it is not yet clear why RNAi-mediated knockdown of GADD45A reduces denervation-induced atrophy in the TA, but *Gadd45a* gene deletion does not reduce denervation-induced atrophy in the gastrocnemius. Some possible explanations include inherent differences in the TA and gastrocnemius muscles and/or technical differences between acute RNAi-mediated knockdown and lifelong gene deletion methods, which may generate different off-target consequences and compensatory changes. It is also important to note that the molecular mechanisms of muscle fiber atrophy can vary based on the underlying cause of muscle atrophy and on the severity and duration of that stress, and every known molecular mediator of muscle atrophy has been dissociated from the pathogenesis of muscle atrophy caused by at least one atrophy-inducing condition (10). In some cases, mechanisms are dissociated because they do not occur during a particular atrophy-inducing condition, and in other cases, mechanisms do occur, but they are nonessential for muscle atrophy in that specific context, due to redundancy or due to the acquisition of new roles or functions in that specific context (10). Thus, additional studies are still needed to better understand the degree to which GADD45A is required for muscle atrophy in contexts that have been investigated to some extent (e.g., muscle denervation, limb immobilization) and in other clinically important contexts (e.g., advanced age, critical illness, diabetes, cancer, renal failure, heart failure, chronic pulmonary disease, spinal cord injury, weight loss medications and surgeries, Cushing syndrome, chronic infections).

In summary, the current study identifies GADD45A as a molecular mediator of muscle weakness, reduced specific force, impaired exercise capacity, and several cellular changes that often occur during muscle atrophy, including loss of skeletal muscle mitochondria, selective atrophy of glycolytic fibers, and paradoxical expression of oxidative MyHCs in the face of mitochondrial loss. These results provide potentially important insights into the pathogenesis of skeletal muscle atrophy, a complex and debilitating condition that affects hundreds of millions of people around the world.

## Methods

### Mouse strains and animal care.

*HSA-Lox-STOP-Lox-Gadd45a* mice were generated at Cyagen and contained a transgene with the skeletal muscle–specific promoter (human *ACTA1* [*HSA*] promoter), a *Lox-STOP-Lox* cassette, and an epitope-tagged mouse *Gadd45a* cDNA inserted into the mouse *ROSA26* locus. GADD45A-mTg mice were generated by crossing homozygous *HSA-Lox-STOP-Lox-Gadd45a* mice to heterozygous *HSA-MerCreMer* mice, which express a tamoxifen-inducible Cre recombinase construct (MerCreMer) under control of the *HSA* promoter (25). *HSA-MerCreMer* mice were obtained from The Jackson Laboratory (strain 025750). Control mice in the GADD45A-mTg experiments were littermates who possessed the *HSA-Lox-STOP-Lox-Gadd45a* transgene but no *HSA-MerCreMer* transgene. All genetically modified mice (*HSA-Lox-STOP-Lox-Gadd45a*, *HSA-MerCreMer*, and progeny) were backcrossed at least 5 times into the C57BL/6J background. Male C57BL/6 mice were obtained from Charles River Laboratories at 6–10 weeks of age and used for experiments within 2 weeks of their arrival. All animals were housed in colony cages at 21°C with 12-hour light/12-hour dark cycles and with ad libitum access to standard chow and water throughout the study. Standard chow was Harlan Teklad formula 7013 at the University of Iowa and PicoLab Rodent 5053 at Mayo Clinic. Euthanasia methods were approved by the Panel on Euthanasia of the American Veterinary Medical Association. Animals were euthanized by CO_2_ exposure (flow rate of 4 L/min) until breathing stopped for a period of 1 minute, and euthanasia was confirmed by decapitation.

### Analyses of mouse strength, exercise capacity, and specific force.

Forelimb grip strength was determined with a grip strength meter equipped with a triangular pull bar (Columbus Instruments), as described previously (44). Endurance exercise capacity on an accelerating motor-driven open treadmill with a shock grid (Columbus Instruments) was determined as described previously (44); briefly, mice were acclimated to running for 2 days (5 min/d with treadmill speed 5 m/min and treadmill incline at 0%), and then endurance exercise capacity was determined the following day by setting the treadmill incline at 10%, setting the shock grid at 0.2 mA, and treadmill speed at 5 m/min for the first 5 minutes, and increasing treadmill speed by 2 m/min every 2 minutes thereafter. Running was terminated when mice contacted the shock grid for 10 seconds. Ex vivo muscle force generation was determined using an Aurora Scientific 1200A Intact Muscle Test System to determine maximal and specific tetanic force in isolated EDL muscles, as described previously (44).

### Plasmids.

GADD45A plasmid encodes mouse *Gadd45a* under the control of the CMV promoter in the pcDNA3.1(+) vector. Epitope-tagged GADD45A plasmid has been described previously (24) and is identical to the GADD45A plasmid except for the presence of 3 FLAG epitope tags and 2 S-Tags at the N-terminus of GADD45A. The MEKK4ΔN plasmid has been described previously (24) and encodes a start methionine followed by amino acids 1,051–1,597 of mouse MEKK4/MAP3K4 followed by 3 copies of the FLAG epitope tag at the C-terminus, under control of the CMV promoter. The control RNAi plasmid was described previously (7) and encodes emerald green fluorescent protein (EmGFP) and a nontargeting pre-miRNA under bicistronic control of the CMV promoter in the pcDNA6.2GW/EmGFP-miR plasmid (Invitrogen). The MEKK4 RNAi plasmid encodes EmGFP and an artificial pre-miRNA targeting mouse *MEKK4/Map3k4* under bicistronic control of the CMV promoter (24).

### Mouse skeletal muscle transfection.

Mice were anesthetized with 91 mg/kg ketamine and 9.1 mg/kg xylazine, and then the hind limbs were shaved. TA or EDL muscles were then injected with 30 μL of 0.4 U/μL bovine placental hyaluronidase (MilliporeSigma, catalog H4272) resuspended in sterile 0.9% saline using a 30-gauge needle. Two hours later, mice were reanesthetized, and muscles were injected with 30 μL of endotoxin-free plasmid DNA in sterile saline, coated with ultrasound jelly, and then subjected to three 50 ms pulses of 100 V with a 1-second pulse interval using an ECM-830 electroporator (BTX Harvard Apparatus). Following transfection, mice were returned to their cages to resume normal activities for 7–14 days before euthanasia and analyses of transfected muscles.

### Statistics.

Unless otherwise noted, statistical analyses were performed with GraphPad Prism using statistical tests described in the figure legends. All *t* tests were 2-tailed. *P* values, *q* values, and FDR values less than 0.05 were considered significant.

### Study approval.

All animal procedures were approved by the Institutional Animal Care and Use Committees of the University of Iowa and Mayo Clinic. All human participants provided written informed consent, and all human study procedures conformed to the principles of the Declaration of Helsinki and were approved by the Mayo Foundation Institutional Review Board (Rochester, Minnesota, USA) or the University of Texas Medical Branch Institutional Review Board (Galveston, Texas, USA). The human studies were registered under Clinical Trial Numbers NCT03350906, NCT04151901, and NCT02103842.

### Data availability.

Values for all data points in the figures and supplemental figures are available in the Supporting Data Values file in the supplement. All other data are available in supplemental tables in the supplement.

Additional methods used in this study may be found in the Supplemental Methods section.

## Author contributions

GRM, MJM, HEK, ZCR, CJH, SC, ZDVR, SPK, ATM, JMD, IRL, and SME performed experiments. GRM, MJM, HEK, ZCR, PMV, MDS, SC, ZDVR, SPK, JIS, KAN, CEG, VAL, ARJ, BRR, KSN, IRL, SME, and CMA performed data analysis and interpretation. GRM, SME, and CMA wrote the manuscript. All authors edited and approved the manuscript.

## Supplementary Material

Supplemental data

Supplemental table 1

Supplemental table 2

Supplemental table 3

Supplemental table 4

Supplemental table 5

Supplemental table 6

Supplemental table 7

Supplemental table 8

Supporting data values

## Version 1 10/10/2023

In-Press Preview

## Version 2 11/22/2023

Electronic publication

## References

[1] Larsson L (2019). Sarcopenia: aging-related loss of muscle mass and function. Physiol Rev.

[2] Sartori R (2021). Mechanisms of muscle atrophy and hypertrophy: implications in health and disease. Nat Commun.

[3] Ebert SM (2019). Skeletal muscle atrophy: discovery of mechanisms and potential therapies. Physiology (Bethesda).

[4] Ebert SM (2022). Biology of activating transcription factor 4 (ATF4) and its role in skeletal muscle atrophy. J Nutr.

[5] Sacheck JM (2007). Rapid disuse and denervation atrophy involve transcriptional changes similar to those of muscle wasting during systemic diseases. FASEB J.

[6] Ebert SM (2010). The transcription factor ATF4 promotes skeletal myofiber atrophy during fasting. Mol Endocrinol.

[7] Ebert SM (2012). Stress-induced skeletal muscle Gadd45a expression reprograms myonuclei and causes muscle atrophy. J Biol Chem.

[8] Ebert SM (2015). Identification and small molecule inhibition of an activating transcription factor 4 (ATF4)-dependent pathway to age-related skeletal muscle weakness and atrophy. J Biol Chem.

[9] Miller MJ The transcription regulator ATF4 is a mediator of skeletal muscle aging. Geroscience.

[10] Ebert SM (2020). Activating transcription factor 4 (ATF4) promotes skeletal muscle atrophy by forming a heterodimer with the transcriptional regulator C/EBPβ. J Biol Chem.

[11] Welle S (2003). Gene expression profile of aging in human muscle. Physiol Genomics.

[12] Welle S (2004). Skeletal muscle gene expression profiles in 20-29 year old and 65-71 year old women. Exp Gerontol.

[13] Edwards MG (2007). Gene expression profiling of aging reveals activation of a p53-mediated transcriptional program. BMC Genomics.

[14] Gonzalez de Aguilar JL (2008). Gene profiling of skeletal muscle in an amyotrophic lateral sclerosis mouse model. Physiol Genomics.

[15] Banduseela VC (2009). Gene expression and muscle fiber function in a porcine ICU model. Physiol Genomics.

[16] Llano-Diez M (2011). Muscle wasting and the temporal gene expression pattern in a novel rat intensive care unit model. BMC Genomics.

[17] Hulmi JJ (2012). Altered REDD1, myostatin, and Akt/mTOR/FoxO/MAPK signaling in streptozotocin-induced diabetic muscle atrophy. Am J Physiol Endocrinol Metab.

[18] Bongers KS (2013). Skeletal muscle denervation causes skeletal muscle atrophy through a pathway that involves both Gadd45a and HDAC4. Am J Physiol Endocrinol Metab.

[19] Ibebunjo C (2013). Genomic and proteomic profiling reveals reduced mitochondrial function and disruption of the neuromuscular junction driving rat sarcopenia. Mol Cell Biol.

[20] Fox DK (2014). p53 and ATF4 mediate distinct and additive pathways to skeletal muscle atrophy during limb immobilization. Am J Physiol Endocrinol Metab.

[21] Milan G (2015). Regulation of autophagy and the ubiquitin-proteasome system by the FoxO transcriptional network during muscle atrophy. Nat Commun.

[22] Llano-Diez M (2019). RNA-sequencing reveals altered skeletal muscle contraction, E3 ligases, autophagy, apoptosis, and chaperone expression in patients with critical illness myopathy. Skelet Muscle.

[23] Ehmsen JT (2021). GADD45A is a protective modifier of neurogenic skeletal muscle atrophy. JCI Insight.

[24] Bullard SA (2016). Gadd45a protein promotes skeletal muscle atrophy by forming a complex with the protein kinase MEKK4. J Biol Chem.

[25] McCarthy JJ (2012). Inducible Cre transgenic mouse strain for skeletal muscle-specific gene targeting. Skelet Muscle.

[26] Sartori R (2009). Smad2 and 3 transcription factors control muscle mass in adulthood. Am J Physiol Cell Physiol.

[27] Schiaffino S, Reggiani C (2011). Fiber types in mammalian skeletal muscles. Physiol Rev.

[28] Short KR (2005). Decline in skeletal muscle mitochondrial function with aging in humans. Proc Natl Acad Sci U S A.

[29] Lanza IR (2008). Endurance exercise as a countermeasure for aging. Diabetes.

[30] Lanza IR (2012). Chronic caloric restriction preserves mitochondrial function in senescence without increasing mitochondrial biogenesis. Cell Metab.

[31] Porter C (2015). Mitochondrial respiratory capacity and coupling control decline with age in human skeletal muscle. Am J Physiol Endocrinol Metab.

[32] Robinson MM (2017). Enhanced protein translation underlies improved metabolic and physical adaptations to different exercise training modes in young and old humans. Cell Metab.

[33] Hyatt H (2019). Mitochondrial dysfunction induces muscle atrophy during prolonged inactivity: a review of the causes and effects. Arch Biochem Biophys.

[34] Akimoto T (2005). Exercise stimulates Pgc-1alpha transcription in skeletal muscle through activation of the p38 MAPK pathway. J Biol Chem.

[35] Pogozelski AR (2009). p38gamma mitogen-activated protein kinase is a key regulator in skeletal muscle metabolic adaptation in mice. PLoS One.

[36] Short KR (2005). Changes in myosin heavy chain mRNA and protein expression in human skeletal muscle with age and endurance exercise training. J Appl Physiol (1985).

[37] Sandri M (2006). PGC-1alpha protects skeletal muscle from atrophy by suppressing FoxO3 action and atrophy-specific gene transcription. Proc Natl Acad Sci U S A.

[38] Reed SA (2012). Inhibition of FoxO transcriptional activity prevents muscle fiber atrophy during cachexia and induces hypertrophy. FASEB J.

[39] Nilwik R (2013). The decline in skeletal muscle mass with aging is mainly attributed to a reduction in type II muscle fiber size. Exp Gerontol.

[40] Baehr LM (2017). Muscle-specific and age-related changes in protein synthesis and protein degradation in response to hindlimb unloading in rats. J Appl Physiol (1985).

[41] Hunt LC (2021). Integrated genomic and proteomic analyses identify stimulus-dependent molecular changes associated with distinct modes of skeletal muscle atrophy. Cell Rep.

[42] Cai D (2004). IKKbeta/NF-kappaB activation causes severe muscle wasting in mice. Cell.

[43] Nair KS (2005). Aging muscle. Am J Clin Nutr.

[44] Dyle MC (2014). Systems-based discovery of tomatidine as a natural small molecule inhibitor of skeletal muscle atrophy. J Biol Chem.

[45] Sanchez R (2010). Solution structure of human growth arrest and DNA damage 45alpha (Gadd45alpha) and its interactions with proliferating cell nuclear antigen (PCNA) and Aurora A kinase. J Biol Chem.

