# Supplementary material for: GADD45A is a mediator of mitochondrial loss, atrophy, and weakness in skeletal muscle
Source: JCI Insight. 2023 Nov 22;8(22):e171772. doi: 10.1172/jci.insight.171772 (PMC10721312; doi:10.1172/jci.insight.171772)
Supplement: Supplemental data [file jciinsight-8-171772-s023.pdf]

SUPPLEMENTAL MATERIAL

Supplemental Table 1. TMT-mass spectrometry data from quadriceps muscles of 15-month-old male littermate control and GADD45A mTg mice. Data include raw intensities, log2 fold changes, P-values, and FDR values of 5206 identified proteins.

Supplemental Table 2. Total raw intensity values for peptides unique to MyHC-2B, MyHC-2X, MyHC-2A and MyHC-slow. Data are from quadriceps muscles of 15-month-old male littermate control and GADD45A mTg mice.

Supplemental Table 3. Reactome pathway enrichment analysis of proteomic data from quadriceps muscles of 15-month-old male littermate control and GADD45A mTg mice.

Supplemental Table 4. TMT-mass spectrometry data for mitochondrial proteins contained in the mouse MitoCarta 3.0 database. Data are from quadriceps muscles of 15-month-old male littermate control and GADD45A mTg mice.

Supplemental Table 5. TMT-mass spectrometry data from tibialis anterior muscles of 8-week-old male C57BL/6 mice transfected with either empty plasmid (Control) or plasmid encoding MEKK4ΔN. Data include raw intensities, log2 fold changes, P-values, and FDR values of 6870 identified proteins.

Supplemental Table 6. Total raw intensity values for peptides unique to MyHC-2B, MyHC-2X, MyHC-2A and MyHC-slow. Data are from tibialis anterior muscles of 8-week-old male C57BL/6 mice transfected with either empty plasmid (Control) or plasmid encoding MEKK4ΔN.

Supplemental Table 7. Reactome pathway enrichment analysis of proteomic data from tibialis anterior muscles of 8-week-old male C57BL/6 mice transfected with either empty plasmid (Control) or plasmid encoding MEKK4ΔN.

Supplemental Table 8. Eighteen highly abundant skeletal muscle proteins that were added to the Swiss-Prot FASTA mouse database (Taxon ID: 10090, Uniprot release 2021_03) for searches of the raw mass spectrometry files in this study.

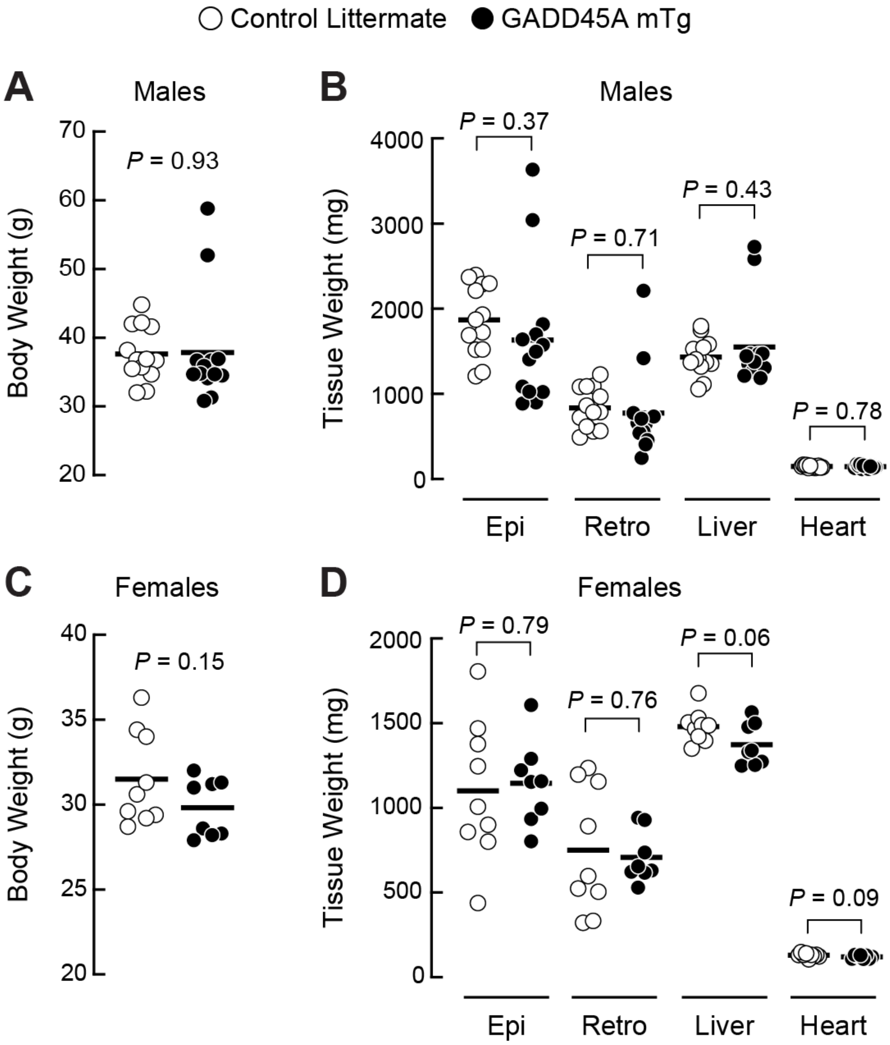

Supplemental Figure 1. Body weights and weights of bilateral epidydimal fat pads (Epi), bilateral retroperitoneal fat pads (Retro), liver, and heart in male and female littermate control and GADD45A mTg mice. Male mice were 15-months old, and female mice were 17-months old. Each circle represents one animal, and horizontal bars denote the means. *P*-values were determined with unpaired two-tailed t-tests.

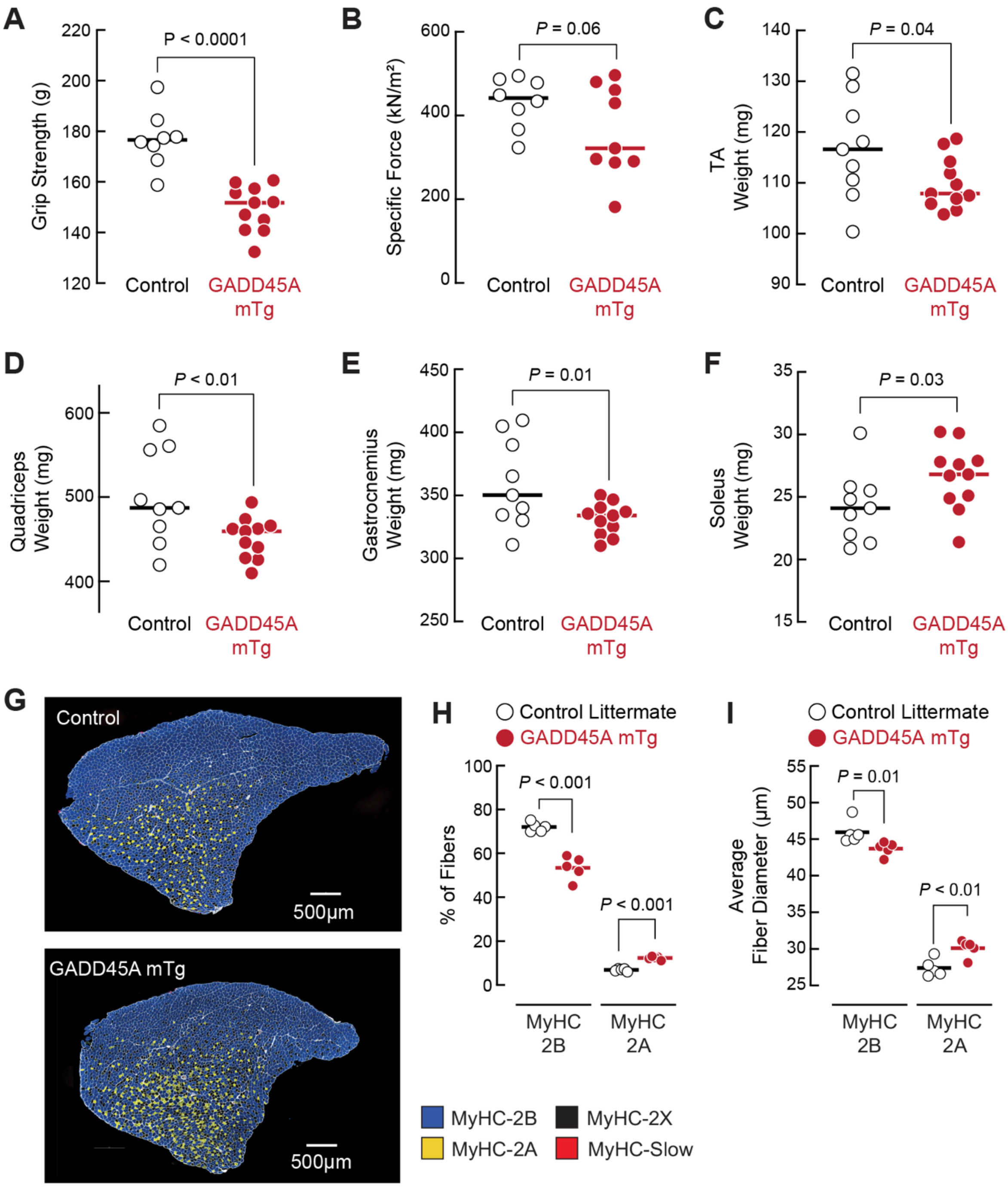

Supplemental Figure 2. 8-month-old GADD45A mTg mice exhibit reduced skeletal muscle function and mass, atrophy of skeletal muscle fibers that express MyHC-2B, and increased size and relative amount of skeletal muscle fibers that express MyHC-2A. (A-F) 8-month-old male littermate control and GADD45A mTg mice were subjected to analyses of grip strength (A), ex vivo specific force in

extensor digitorum longus muscles (B), and wet weights of bilateral tibialis anterior (TA) muscles (C), quadriceps femoris muscles (D), gastrocnemius muscles (E), and soleus muscles (F). Each circle represents the value from one animal, and horizontal bars indicate mean values. *P*-values were determined with unpaired one-tailed t-tests. (G-I) TA muscles from 8-month-old male littermate control and GADD45A mTg mice were sectioned and subjected to immunofluorescence microscopy using antibodies targeting laminin (white), MyHC-slow (red), MyHC-2A (yellow), and MyHC-2B (blue). Muscle fibers without MyHC-slow, MyHC-2A, and MyHC-2B were assigned to the unstained Myh isoform (MyHC-2X). (G) Representative images. (H) Relative amounts of muscle fibers expressing MyHC-2B or MyHC-2A. (I) Average minimal Feret diameters of muscle fibers expressing MyHC-2B or MyHC-2A In H-I, each data point represents the mean value from one muscle, horizontal bars indicate mean values from each group, and *P*-values were determined with unpaired one-tailed t-tests.

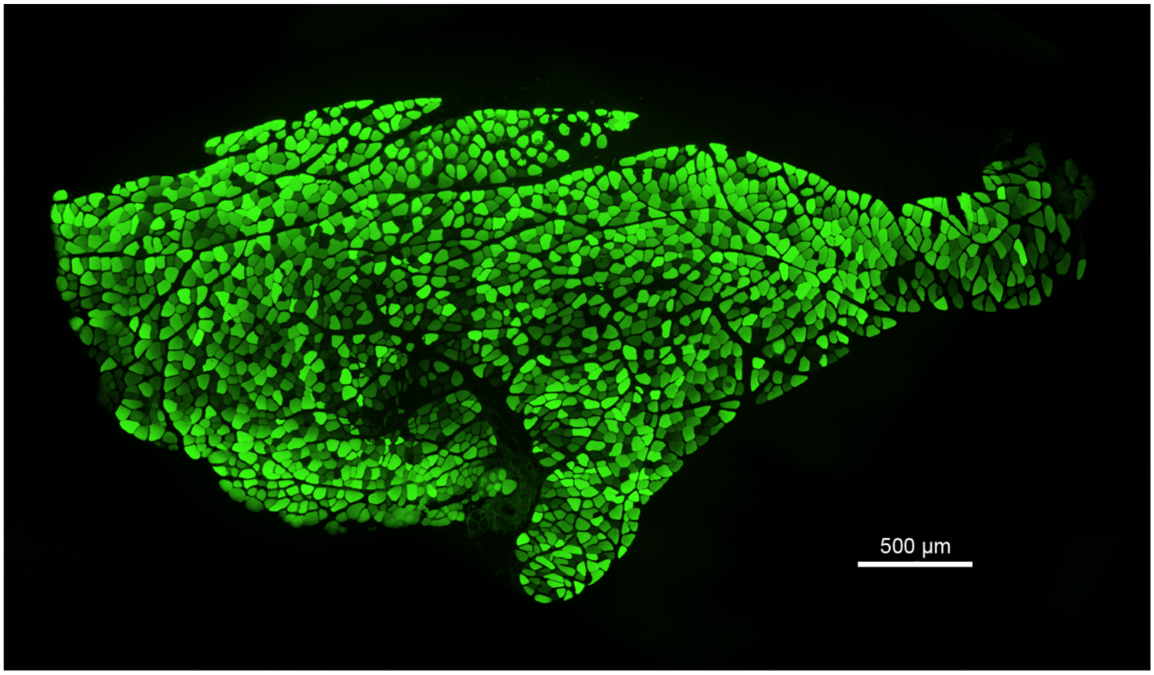

Supplemental Figure 3. Representative transfection efficiency with the in vivo skeletal muscle transfection method used in this study. Tibialis anterior muscle of 8-week-old male C57BL/6 mice was transfected with 5 µg reporter plasmid containing a cDNA containing enhanced green fluorescent protein (eGFP) under control of the CMV promoter. Seven days later, transfected muscle was harvested, fixed, cryosectioned, and subjected to fluorescence microscopy for reporter plasmid expression (eGFP, which appears green), as described previously (1). Image shows the entire muscle cross-section. Transfection efficiency was > 70%, which is consistently observed with this method.

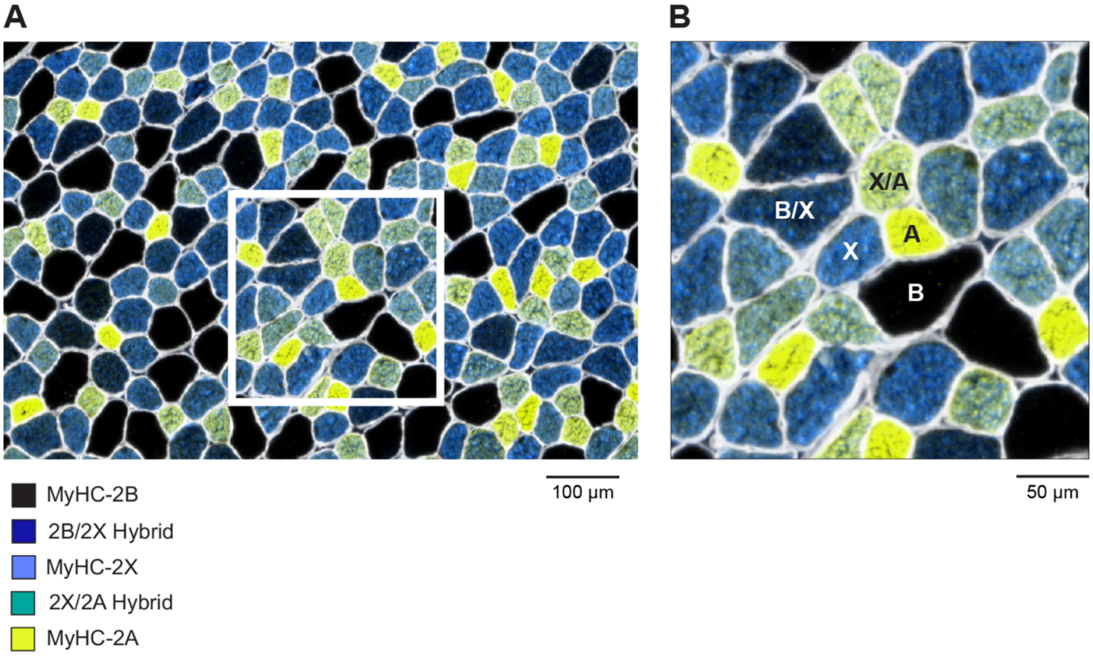

Supplemental Figure 4. Evidence of MyHC-2B/2X and MyHC-2X/2A hybrid fibers in skeletal muscle with GADD45A overexpression. Tibialis anterior muscle of 8-week-old male C57BL/6 mice was transfected with 2.5 µg plasmid encoding mouse GADD45A. Seven days later, transfected muscle was sectioned and subjected to immunofluorescence microscopy using antibodies targeting laminin (white), MyHC-slow (red), MyHC-2A (yellow), and MyHC-2X (light blue). Muscle fibers without MyHC-slow, MyHC-2A, and MyHC-2X were assigned to the unstained Myh isoform (MyHC-2B). (A) Representative image. (B) Higher magnification of the region denoted by white box in (A), showing laminin (white), MyHC-2A fibers (yellow), MyHC-2X fibers (light blue), MyHC-2B fibers (black), likely MyHC-2B/2X hybrid fibers (dark blue), and likely MyHC-2X/2A fibers (green). Some of the fibers are labelled with the MyHC isoform(s) that appear to be contained within them.

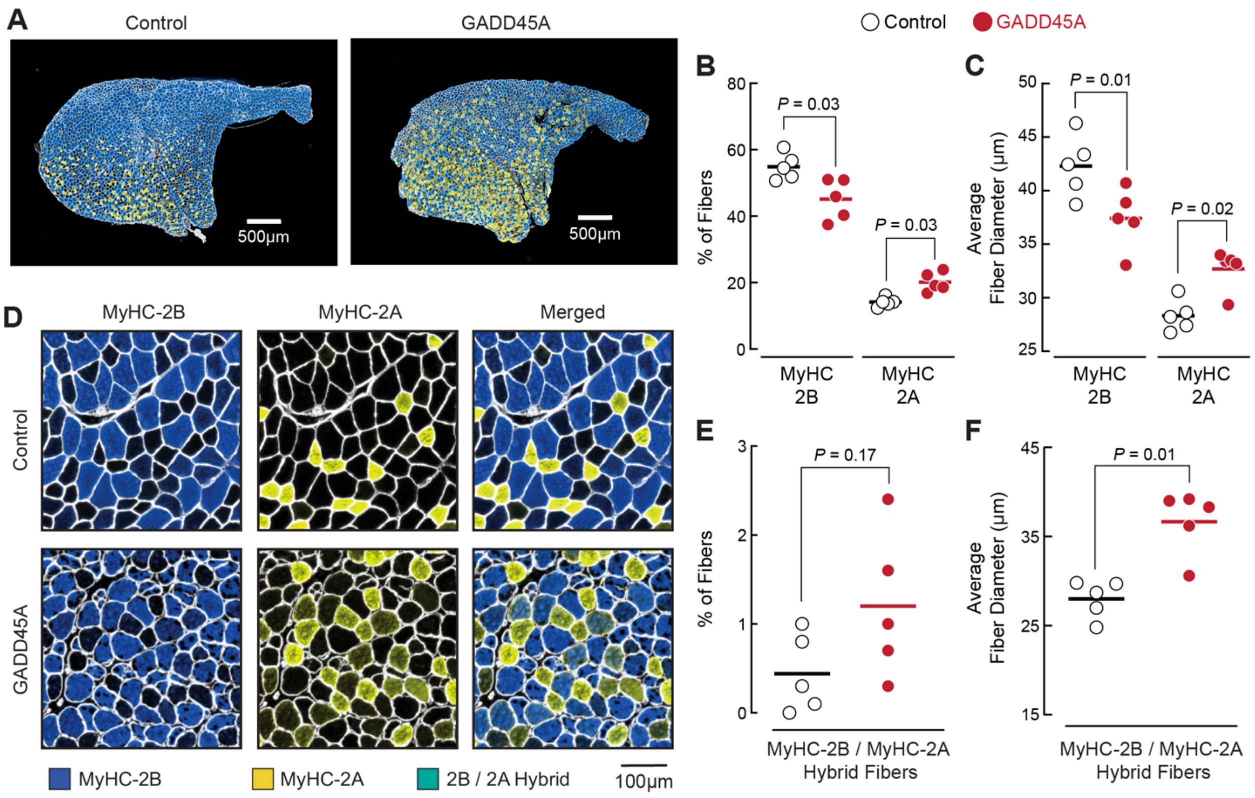

Supplemental Figure 5. Acute expression of GADD45A in skeletal muscle induces atrophy of large, glycolytic MyHC-2B-positive muscle fibers and increases the size and relative amount of small MyHC-2A-positive muscle fibers. Tibialis anterior (TA) muscles of 8-week-old male C57BL/6 mice were transfected with plasmid DNA. One TA per mouse was transfected with 2.5 µg empty plasmid (Control), and the contralateral TA in each mouse was transfected with 2.5 µg plasmid encoding the same N-terminal epitope-tagged mouse GADD45A used in GADD45A mTg mice. Seven days later, bilateral TAs were sectioned and subjected to immunofluorescence microscopy using antibodies targeting laminin, MyHC-2B, MyHC-2A, and MyHC-slow. (A) Representative images of entire TA muscle. MyHC-2B staining is blue, MyHC-2A staining is yellow, and MyHC-slow staining is red. (B) Relative amounts of muscle fibers expressing MyHC-2B or MyHC-2A. (C) Average minimal Feret diameters of muscle fibers expressing MyHC-2B or MyHC-2A. (D) Higher magnification images showing laminin staining (white), MyHC-2B staining (blue), MyHC-2A staining (yellow), and fibers expressing both MyHC-2B and MyHC-2A (green). (E) Relative amounts of muscle fibers expressing both MyHC-2B and MyHC-2A.

(F) Average minimal Feret diameters of muscle fibers expressing both MyHC-2B and MyHC-2A. In B-C and E-F, each data point represents the mean value from one muscle, horizontal bars indicate mean values from each group, and *P*-values were determined with paired two-tailed t-tests.

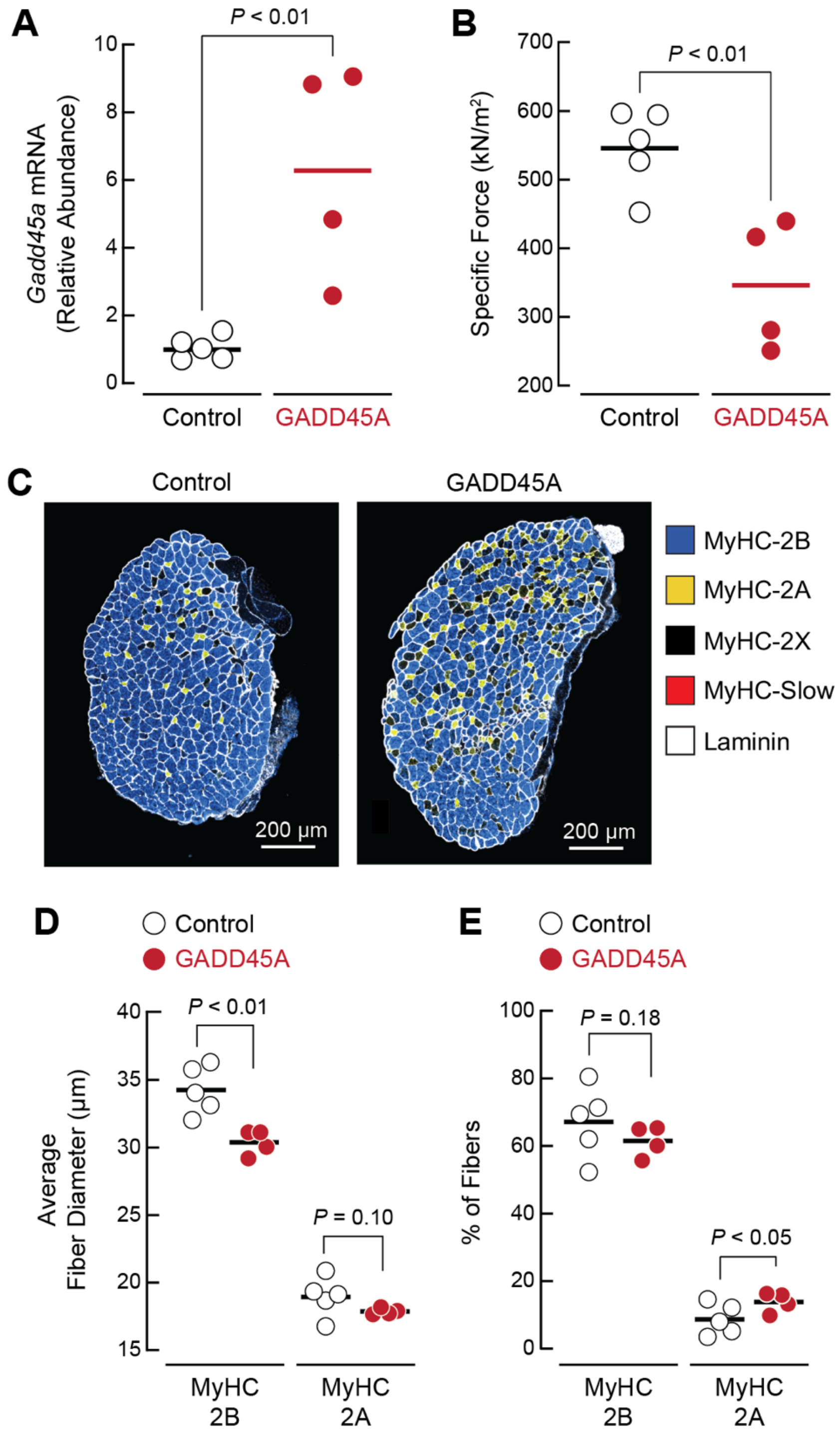

Supplemental Figure 6. Acute expression of GADD45A in the extensor digitorum longus (EDL) muscle decreases specific force, induces atrophy of large glycolytic muscle fibers, and increases the relative amount of small MyHC-2A-positive muscle fibers. EDL muscles of 10-week-old male

C57BL/6 mice were transfected with 2.5 μg empty plasmid (Control) or 2.5 μg plasmid encoding mouse GADD45A. Seven days later, transfected muscles were harvested and subjected to (A) qPCR analysis of *Gadd45a* mRNA levels, (B) analysis of ex vivo specific force, and (C-E) immunofluorescence microscopy using antibodies targeting laminin, MyHC-2B, MyHC-2A, and MyHC-slow. (C) Representative images. (D) Average minimal Feret diameters of muscle fibers expressing MyHC-2B or MyHC-2A. (E) Relative amounts of muscle fibers expressing MyHC-2B or MyHC-2A. In A, B, D and E, each data point represents the value from one muscle, horizontal bars indicate mean values, and *P*-values were determined with one-tailed t-tests.

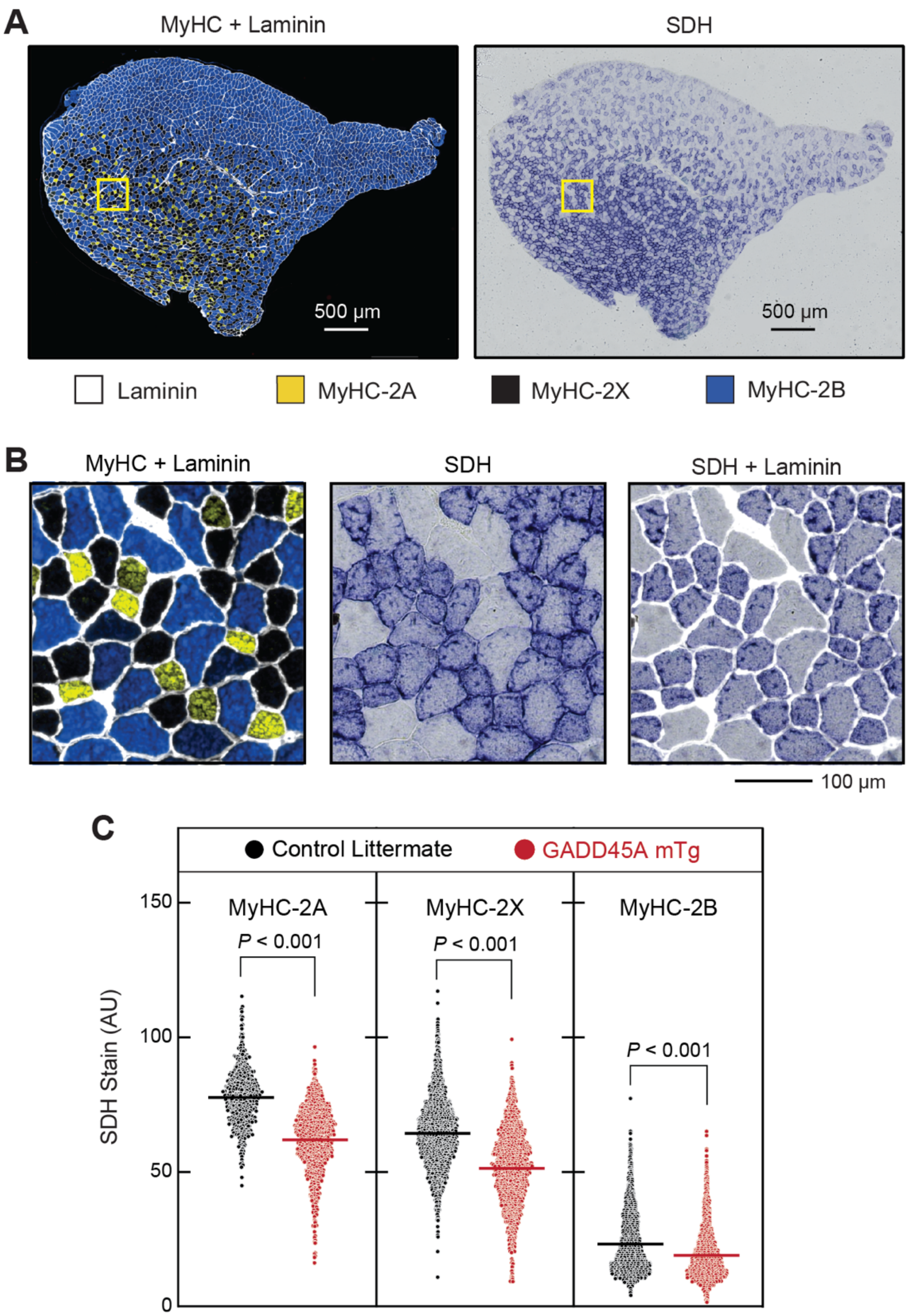

Supplemental Figure 7. GADD45A reduces oxidative capacity in glycolytic and oxidative skeletal muscle fibers. Tibialis anterior muscles from 15-month-old male littermate control and GADD45A mTg mice were cryosectioned. Muscle cross-sections were stained for succinate dehydrogenase (SDH)

activity and then labelled with antibodies targeting MyHC isoforms and laminin. Cross-sections were then imaged by brightfield and fluorescence microscopy to capture SDH activity and immunostaining, respectively. Captured images were then overlaid, and SDH activity and MyHC expression were quantitated in every fiber in the muscle, in order to determine SDH activity as a function of MyHC expression. (A) Representative cross-section from a tibialis anterior muscle of a littermate control mouse. MyHC and laminin immunofluorescence is shown on the left, and SDH activity is shown on the right. Images were obtained at low magnification to visualize the entire muscle cross-section. (B) Higher magnification of the regions denoted by yellow boxes in (A). The image on the right merges laminin and SDH staining. (C) Quantification of MyHC expression vs. SDH activity in tibialis anterior muscles of littermate control and GADD45A mTg mice. Each data point represents the value from one muscle fiber, and horizontal bars indicate mean values. Data are from 3 muscles per genotype. *P*-values were determined with unpaired two-tailed t-tests.

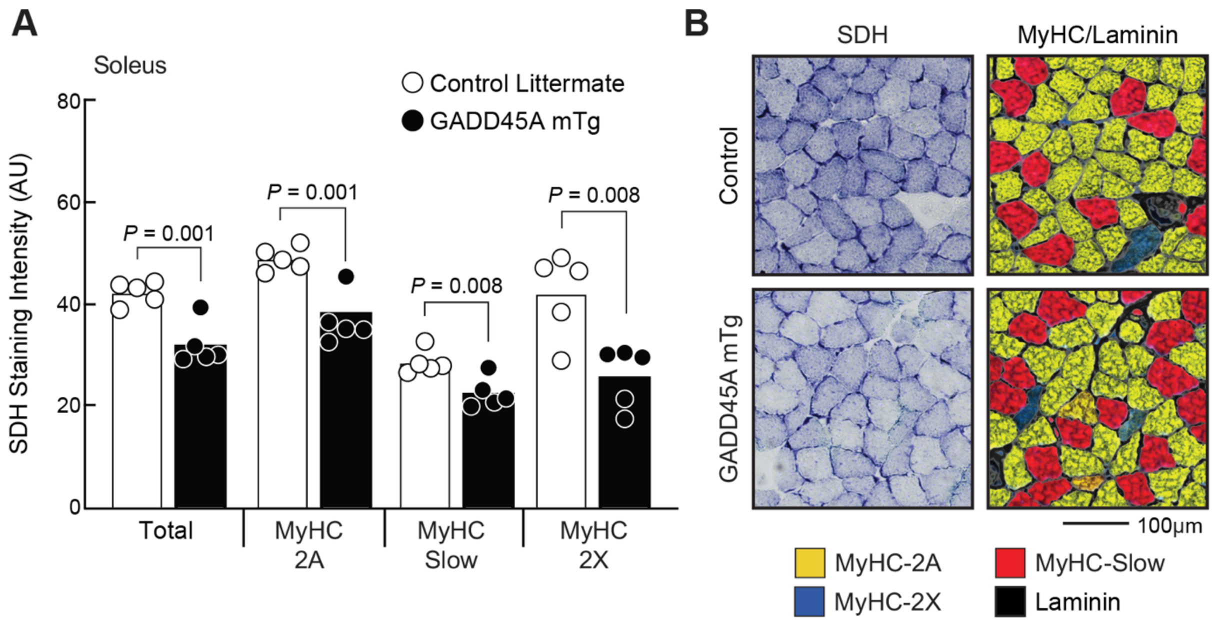

Supplemental Figure 8. GADD45A reduces oxidative capacity in soleus skeletal muscle fibers. Soleus muscles from 15-month-old male littermate control and GADD45A mTg mice were cryosectioned. Muscle cross-sections were stained for succinate dehydrogenase (SDH) activity and then labelled with antibodies targeting MyHC isoforms and laminin. Cross-sections were then imaged by brightfield and fluorescence microscopy to capture SDH activity and immunostaining, respectively. Captured images were then overlaid, and SDH activity and MyHC expression were quantitated in every fiber in the muscle, in order to determine SDH activity as a function of MyHC expression. (A) Quantification of MyHC expression vs. SDH activity. Each data point represents the value from one muscle, bars indicate mean values, and *P*-values were determined with unpaired two-tailed t-tests. (B) Representative SDH and MyHC/laminin stains of soleus cross-sections from littermate control and GADD45A mTg mice.

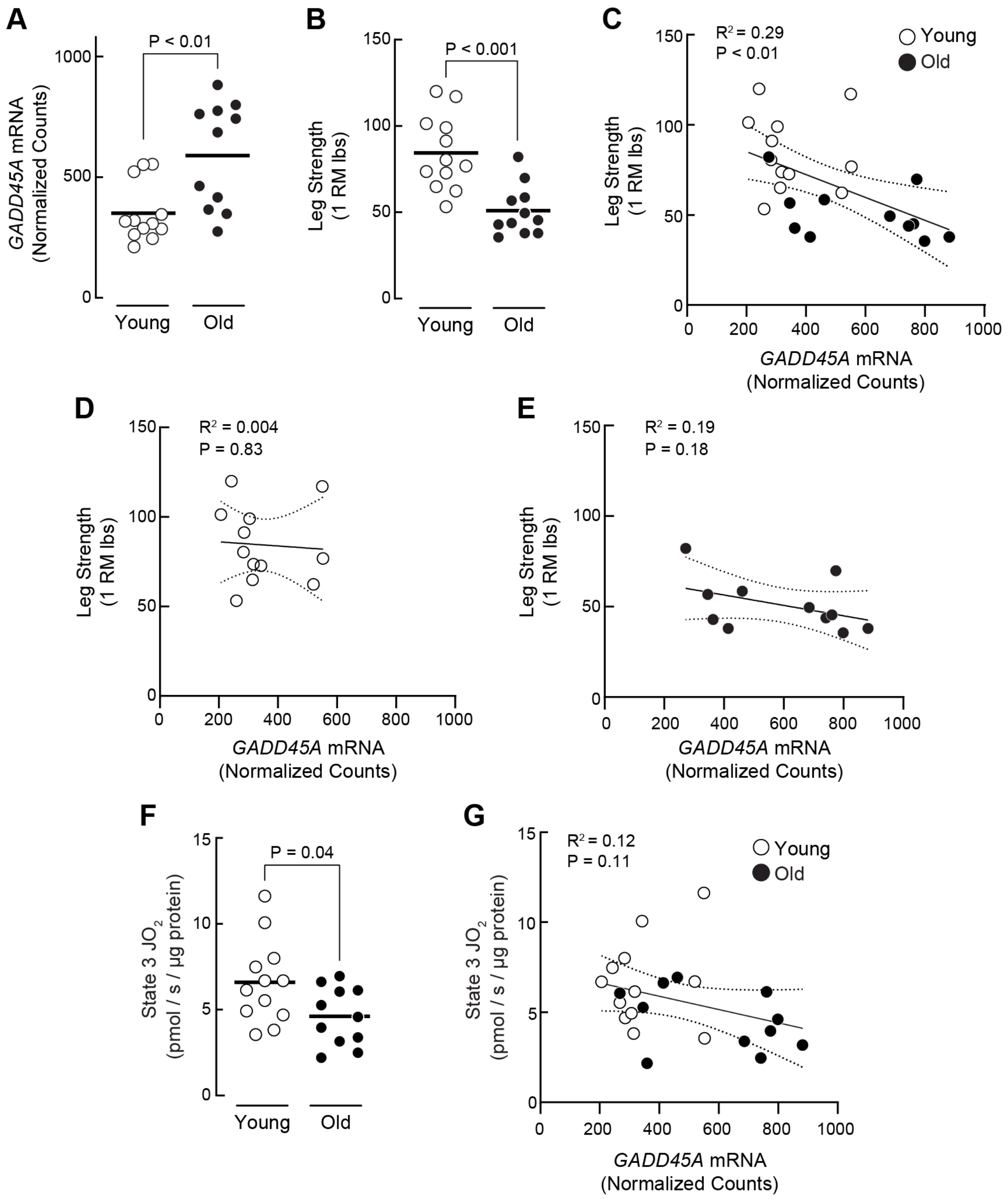

Supplemental Figure 9. Skeletal muscle *GADD45A* expression is associated with muscle weakness in humans. A second, independent group of human subjects (different from the subjects in Figure 9) who were 18- to 30-years-old (young) or 65- to 80-years-old (old) volunteered for a study in which *GADD45A* mRNA levels in the vastus lateralis muscle were assessed by RNA sequencing, maximal leg

strength was assessed via 1 repetition maximum (1RM) leg press (a dynamic movement through a full range of motion of knee extension) and state 3 mitochondrial respiration (JO_2) was assessed in isolated mitochondria from vastus lateralis muscle. (A) *GADD45A* mRNA levels. (B) Maximal leg strength. (C) Correlation of *GADD45A* mRNA levels to maximal leg strength across all subjects, young and old. (D) *GADD45A* mRNA levels vs. maximal leg strength in young subjects. (E) *GADD45A* mRNA levels vs. maximal leg strength in old subjects. (F) State 3 mitochondrial respiration. (G) Correlation of *GADD45A* mRNA levels to state 3 mitochondrial respiration. In A-G, each data point represents the value from one subject. In A, B and F, horizontal bars indicate mean values, and *P*-values were determined with unpaired two-tailed t-tests. In C, D, E and G, Pearson's correlation coefficients and *P*-values were determined with simple linear regressions.

SUPPLEMENTAL METHODS

Mouse genotyping - Mouse tail clips were incubated in 100 µL Direct PCR Lysis Reagent (Tail) (Viagen, catalog no. 102-T) containing 1 µL proteinase K (Qiagen, catalog no. 166041314) at 55 °C for 16 h, followed by a 1-hour incubation at 85 °C. Samples were then centrifuged at 16,000 x *g* for 1 min at 21 °C. One µL of supernatant was used for PCR reaction mixture containing *Taq* DNA polymerase with standard *Taq* Buffer (New England Biolabs, catalog no. M0273) according to the manufacturer's instructions. PCR conditions included an initial denaturation step at 95 °C for 30 s, followed by 40 cycles of a denaturation phase at 95 °C for 30 s, an annealing phase at 57 °C for 30 s, and an extension phase at 68 °C for 1 min. A final extension phase of 68 °C for 5 min was completed before PCR products were separated by electrophoresis on a 2% agarose gel containing ethidium bromide from (Bio-Rad, catalog no. 1610433) and visualized under UV light using an iBright FL1500 imaging system from Invitrogen. Mouse genotypes were confirmed using three sets of primers. Primer set 1 targeted the *ACTA1* promoter and upstream homology arm of the GADD45A transgene and was 5'TACTTATGGTAAAGTAGAGTGGG-3' and 5'-ATCTACCTCGATGGAAAATACTC-3'. Primer set 2 targeted the Cre recombinase transgene and was 5'-AGGTGGACCTGATCATGGAG-3'and 5'-ATACCGGAGATCATGCAAGC-3'. Primer set 3 was used as an internal control to confirm a successful PCR reaction and was 5'-CTAGGCCACAGAATTGAAAGATCT-3'and 5'-GTAGGTGGAAATTCTAGCATCATCC-3'.

Buffers - Buffer A was phosphate-buffered saline (Gibco), pH 7.4, 0.5% Triton X-100, and 5% horse serum. Buffer B was 0.2 M sodium phosphate buffer, pH 7.3, 0.2 M sodium succinate, and 0.5 mg/ml nitro blue tetrazolium. Buffer C was 50 mM Tris, pH 8.0, 150 mM sodium chloride, 10% SDS, 1% NP-40, 0.5% sodium deoxycholate, 1 mM EDTA, complete mini-EDTA-free protease inhibitor, and PhosStop phosphatase inhibitor. Buffer D was 50 mM Tris, pH 7.2, 100 mM potassium chloride, 5 mM

magnesium chloride, 1.8 mM ATP, and 1 mM EDTA. Buffer E (MiR05) was 10 mM potassium dihydrogen phosphate buffer, pH 7.1, 0.5 mM EGTA, 3 mM magnesium chloride hexahydrate, 60 mM potassium lactobionate, 20 mM taurine, 20 mM HEPES, 110 mM sucrose, and 1 g/L fatty acid free BSA.

Quantitative real-time RT-qPCR - Skeletal muscles were placed in 1 ml of TRIzol solution (Thermofisher, catalog no. 15596026) and then homogenized with three 30 s cycles of Precellys Evolution (Bertin Technologies, catalog no. PE000062) homogenizer set at 8200 rpm. RNA was then extracted following the manufactures directions and purified using the Turbo DNA-free kit (Invitrogen, catalog no. AM1907). For qPCR studies, first strand cDNA was synthesized in a 20 µl reaction that contained 2 µg of RNA, random hexamer primers, and components of the High-Capacity cDNA Reverse Transcription Kit (Applied Biosystems, catalog no. 4368814). qPCR analysis of mouse *Gadd45a* and *36B4* mRNA was performed using TaqMan Gene Expression Assays (Applied Biosystems). qPCR was carried out using a QuantStudio 6 Pro Real-Time PCR System (ThermoFisher, catalog no. A43054). All qPCR was performed in triplicate, and the cycle threshold (C_t) values were averaged to give the final results. To analyze the data, we used the ΔC_t method, with the level of *36B4* serving as the invariant control.

Proteomic sample preparation - Freshly excised mouse skeletal muscles were immediately frozen in liquid N_2 and stored at -80 °C. Whole, frozen muscles were then pulverized to powder using a stainless-steel homogenizing apparatus embedded in dry ice, and then powdered tissue was weighed, lysed at 21 °C for 3 seconds in 20x (v/w) Buffer C using a handheld Pellet Pestle Motor (Kontes), and immediately frozen. Samples were then thawed, centrifuged at 20,000 x *g* for 5 min at 21 °C, and clarified supernatant was saved in a new tube. Soluble protein concentration was measured using the Pierce BCA Protein Assay (ThermoFisher, catalog no. 23225), and 200 µg of soluble protein from each sample was digested with trypsin/Lys-C mixture (Promega, catalog no. V5072) at 37 °C for 16 h, using S-Trap Mini columns

from Protifi, per manufacturer's instructions. Resultant peptide isolate concentrations were measured with a quantitative colorimetric peptide assay (Pierce, catalog no. 23275). An aliquot of 100 µg peptides from each replicate sample was dried down in a SpeedVac, reconstituted in 100 mM TEAB (ThermoScientific, catalog no. 90114), and labeled with tandem mass tags (TMT 10-plex) according to manufacturer's specifications (ThermoScientific, catalog no. 90406). Following TMT labeling, equal sample mixing, C18 clean-up, and lyophilization, samples were suspended in 10 mM ammonium formate, pH 9.0, and fractionated via high pH RP-HPLC (Agilent). Peptide separation and fraction was done using a 4.6 x 150 mm x 3.5 µm XBridge C18 column (Waters) over a 90 min gradient from 2 to 40% mobile phase B. Mobile phase A was 10 mM ammonium formate, and mobile phase B was 10 mM ammonium formate in 80% acetonitrile. A total of 96 fractions were collected and subsequently concatenated to 24 fractions. Approximately 3 µg from each fraction was transferred to a sample vial, dried in a SpeedVac, suspended in 0.2% formic acid/0.1% trifluoroacetic acid/0.002% zwittergent 3-16 sample buffer, and subjected to mass spectrometry.

Mass spectrometry - TMT-labeled peptide from each sample was analyzed by nanoflow liquid chromatography electrospray tandem mass spectrometry using a Thermo Ultimate 3000 RSLCnano HPLC autosampler system coupled to an Orbitrap Fusion Lumos mass spectrometer (ThermoScientific). Peptides (500 ng) were loaded on a 33 µl EXP stem trap packed with HALO C18 resin (2.7 µM, 90 Å, Optimize Technologies). Peptides were eluted at a flow rate of 350 nL/min from the trap through a 100 µm x 30 cm PicoFrit column (New Objective) packed in-house with Acclaim Pepmap C18 resin (ThermoFisher). Chromatography was performed using a 2% to 35% gradient of Solvent B over 120 min (Solvent A: 98% water/2% acetonitrile/0.2% formic acid, Solvent B: 80% acetonitrile/10% isopropanol/10% water/0.2% formic acid). The Fusion Lumos mass spectrometer was set to acquire ms1 survey scans from 340-1600 m/z at a resolution of 120,000 (at 200 m/z) with the automatic gain target (AGC) set at 4e5 ions and a maximum ion inject time of 50 msec. Survey scans were followed by ms2

high energy collision dissociation (HCD) set at 38%, resolution of 50,000 (at 200 m/z), an AGC target of 1e5 ions, a maximum ion inject time of 120 msec, and the isolation window set at .07 Da. Dynamic exclusion placed selected ions on an exclusion list for 60 sec.

Proteomic data analysis - Proteome Discoverer v3.0 software was used for database searching and extracting quantitative values for downstream analysis. Mass spectrometry files were searched using the Sequest search engine against a mouse-specific, Swiss-Prot FASTA database (https://www.uniprot.org, Taxon ID: 10090, Uniprot release 2021_03) containing 18 additional highly abundant skeletal muscle proteins listed in Supplemental Table 8. Search settings included precursor mass tolerance 10 ppm, fragment mass tolerance 0.02 Da, variable modification oxidation methionine, and static modifications for carbamidomethyl cysteine and TMT 10-plex labels on lysines and N-termini. Enzyme specificity was set to trypsin, and a maximum of two missed cleavages were allowed. A false discovery rate (FDR) of 1% was required for both proteins and peptides, using a target-decoy approach. Protein data was exported from the Proteome Discoverer result file (*.pdResult) and differential quantification was calculated from reported TMT protein intensities using a generalized linear model in R (2). Briefly, log2-transformed and quantile-normalized intensities were modeled using a Gaussian-linked generalized linear model. An ANOVA test was used to detect differentially expressed proteins between experimental conditions, and the resulting *P*-values were FDR-corrected using the Benjamini-Hochberg procedure. Fractional abundances of MyHC-2B, MyHC-2X, MyHC-2A and MyHC-slow were quantified as described previously (3) using total raw intensity values (non-transformed and non-normalized) for peptides unique to MyHC-2B, MyHC-2X, MyHC-2A or MyHC-slow shown in Tables S2 and S6. In each sample, we calculated the fractional abundances of MyHC-2B, MyHC-2X, MyHC-2A and MyHC-slow relative to the total combined amount of MyHC-2B, MyHC-2A, MyHC-2X and MyHC-slow in that same sample.

Sample preparation for histological analyses - Harvested skeletal muscles were immediately placed in optimal cutting temperature (O.C.T.) compound (Tissue-Tek, catalog no. 4583) and snap frozen using a

Stand-Alone Gentle Jane from Instrumedics Inc. 10 μm sections were taken from muscle mid-bellies using a Cryostar HM525 NX (Epredia). For immunofluorescence staining, cryosections were immersed in acetone for 10 min at -20 °C, rinsed three times with PBS, blocked in Buffer A for 20 min at 21 °C, rinsed three times with PBS, incubated for 1 h at 21 °C in Buffer A supplemented with primary antibodies, rinsed three times with PBS, and incubated for 1 h at 21 °C in Buffer A supplemented with the appropriate secondary antibodies, rinsed three times with PBS, and then mounted in ProLong Gold Antifade reagent (Invitrogen, catalog no. P36930) and overlayed with a coverslip (FisherScientific, catalog no. 12-545k). For immunofluorescence of tibialis anterior and quadriceps muscles, a 1:250 dilution of rabbit polyclonal anti-laminin IgG (Sigma, catalog no. L9393) and a 1:500 dilution of Alexa Fluor 568-conjugated anti-rabbit-IgG (Invitrogen, catalog no. 11011) were used for laminin staining; a 1:10 dilution of mouse monoclonal anti-MyHC-2B IgM (DSHB, BF-F3) and a 1:500 dilution of Alexa Fluor 647-conjugated anti-mouse IgM (Invitrogen, catalog no. A21238) were used for MyHC-2B staining; a 1:25 dilution of mouse monoclonal anti-MyHC-2A IgG1 (DSHB, SC-71) and a 1:500 dilution of Alexa Fluor 488-conjugated anti-mouse IgG1 (Invitrogen, catalog no. A21121) were used for MyHC-2A staining; and a 1:25 dilution of mouse monoclonal anti-MyHC-slow IgG2b (DSHB, BA-F8) and a 1:500 dilution of Alexa Fluor 350-conjugated anti-mouse IgG2b (Invitrogen, catalog no. A21140) were used for MyHC-slow staining. For immunofluorescence of soleus muscles, a 1:250 dilution of rabbit polyclonal anti-laminin IgG (Sigma, catalog no. L9393) and a 1:500 dilution of Alexa Fluor 568-conjugated anti-rabbit-IgG (Invitrogen, catalog no. 11011) were used for laminin staining; a 1:10 dilution of mouse monoclonal anti-MyHC-2X IgM (DSHB, 6H1) and a 1:500 dilution of Alexa Fluor 647-conjugated anti-mouse IgM (Invitrogen, catalog no. A21238) were used for MyHC-2X staining; a 1:25 dilution of mouse monoclonal anti-MyHC-2A IgG1 (DSHB, SC-71) and a 1:500 dilution of Alexa Fluor 488-conjugated anti-mouse IgG1 (Invitrogen, catalog no. A21121) were used for MyHC-2A staining; and a 1:25 dilution of mouse monoclonal anti-MyHC-slow IgG2b (DSHB, BA-F8) and a 1:500 dilution of Alexa Fluor 350-conjugated anti-mouse IgG2b (Invitrogen, catalog no. A21140) were used for MyHC-

slow staining. For succinate dehydrogenase (SDH) staining, cryosections were rinsed three times with PBS, incubated in Buffer B for 30 min at 37 °C, rinsed three times with PBS, mounted in Prolong Gold Antifade reagent, and overlayed with a coverslip. For co-staining of SDH and MyHC isoforms (as in Figs. 6, S6, and S7), cryosections were first stained for SDH, and then labelled with antibodies targeting MyHC isoforms and laminin. Investigators performing these and other assays in this study, including but not limited to SDH staining, were blinded to sample identification.

Image analysis - All histological sections were imaged with 10X or 15X objectives on a Nikon Eclipse Ti2-U inverted microscope equipped with Nikon Elements Ar software package and DAPI (catalog no. 96370), GFP (catalog no. 96732), TRITC (catalog no.96374) and Cy5 (catalog no. 96376) filter cubes from IDEX Health and Science (Rochester, NY). Image analysis was performed using the Nikon Elements General Analysis (GA3) software using the homogenous area function. For MyHC identification, fluorescence intensity thresholds for each channel were determined as a fixed percentage of the maximal average pixel intensity identified for a given MyHC, and the same thresholds were consistently used across all experimental conditions. Muscle fibers with MyHC intensities above the set threshold were classified as positively staining, while those below the threshold were classified as negatively staining. Muscle fibers that lacked threshold fluorescence for all stained MyHC isoforms were assigned to the unstained MyHC isoform (MyHC-2X for TA and quadriceps, MyHC-2B for soleus). Muscle fibers that exceeded the fluorescence intensity threshold for multiple MyHC isoforms were classified as hybrid fibers. The number and size of fibers expressing specific MyHC isoforms was determined by merging MyHC and laminin images and then using laminin immunofluorescence to determine the total number of fibers per cross-section, minimum Feret diameter of each fiber, and the mean pixel intensity of each MyHC fluorescence channel within each fiber. For quantification of SDH activity as a function of MyHC expression (as in Figs. 6, S7, and S8), cross-sections were imaged by brightfield and fluorescence microscopy to capture SDH activity and immunostaining, respectively. Captured images were then overlaid, and SDH activity and MyHC expression were quantitated in every

fiber in the muscle. Measurements of total SDH activity (as in Fig. 7) were obtained by quantitating SDH staining in entire muscle cross-sections.

Mitochondrial function - Mitochondria were isolated from whole mouse tibialis anterior muscles and from ~60 mg of human vastus lateralis biopsy samples, as previously described (4). Briefly, muscle samples were harvested, weighed, immediately placed into 1 ml of ice-cold Buffer D, treated with *Bacillus Licheniformis* protease (5.66 mg of 10.66 U/mg per 1 ml of Buffer D), homogenized using a Potter-Elvehjem tissue grinder, and centrifuged at 720 x *g* for 5 min at 4 °C. The supernatant was then transferred to a 1.5 ml microfuge tube and centrifuged at 10,000 x *g* for 5 min at 4 °C to pellet and isolate the mitochondrial fraction, which was resuspended in Buffer E. Aliquots of isolated mitochondria (50 µl from mouse muscle and 90 µl from human muscle) were then placed in an Oxygraph high-resolution respirometer (Oroboros Instruments, Innsbruck, Austria) containing Buffer E. Oxygen consumption (JO_2) was assessed using a stepwise protocol that involved the sequential addition of substrates and inhibitors to assess State 2 respiration (10 mM glutamate, 2 mM malate, 10 mM succinate), State 3 respiration through complexes I and II (5 mM ADP), and State 3 respiration through complex II alone (0.5 µM rotenone). Respiration was normalized to tissue weight and to mitochondrial protein content, which was determined using the Pierce BCA Protein Assay (ThermoFisher, catalog no. 23225).

Human aging studies - Data came from two independent human aging cohort studies of generally healthy individuals without known chronic debilitating disease (5, 6). The studies performed at Mayo Clinic and registered under Clinical Trial Numbers NCT03350906 and NCT02103842. In both studies, individuals were excluded for diabetes or fasting plasma glucose > 126 mg/dL, untreated thyroid disease, anemia (hemoglobin < 11 g/dL for females and <12 g/dL for males), active coronary artery disease, renal insufficiency (serum creatinine > 1.5 mg/dL), liver disease (aspartate aminotransferase > 144 IU/L or alanine aminotransferase > 165 IU/L), blood clotting disorders, or substance abuse. Individuals taking

metformin, insulin, or tricyclic antidepressants were also excluded. Study participants underwent percutaneous biopsy of the vastus lateralis muscle using a modified Bergstrom needle under local anesthetic (2% lidocaine) and sterile conditions (7). Muscle tissue was immediately frozen in liquid nitrogen and transferred to storage at -80°C. In the three days preceding the biopsy, participants were provided standardized meals and instructed to refrain from vigorous physical activity. Snap-frozen muscle samples were pulverized into a fine powder, and total RNA was extracted and purified using the RNeasy Fibrous Tissue Kit (Qiagen). RNA purity and concentration were assessed using a spectrophotometer (Nanodrop). Isolated RNA was submitted to the Mayo Clinic Genome Analysis Core Laboratory for RNA sequencing, as previously described (8). RNA libraries were prepared according to the manufacturer's instructions for the TruSeq RNA Sample Prep Kit v2 (Illumina). Libraries were loaded onto paired end flow cells according to the Illumina cBot and cBot Paired end Cluster Kit v3 protocol. Flow cells were sequenced as paired end reads on an Illumina HiSeq 2000 using the TruSeq SBS Sequencing Kit v3 and HCS v2.0.12 data collection software. Base-calling was performed using Illumina's RTA v1.17.21.3. The RNA-Seq data was analyzed using the Mayo Clinic Bioinformatics Core pipeline, MAP-RSeq v1.2.1 (9). Normalization and differential expression analysis was carried out using the edgeR 2.6.2 software package (R Foundation). In the smaller aging study (shown in Fig. S9), single-leg knee extensor strength, defined as the unilateral 1-repetition maximum (1-RM), was determined on two separate visits under investigator guidance (8). Participants were habituated to the exercise and completed a warm-up consisting of 10 repetitions at a light weight. Participants then completed 2 sets of up to 10 repetitions, separated by 3 min of rest. The 1-RM was calculated from the average of the two sets using the formula $1RM = \omega \cdot (1+r/30)$, where ω is the weight in arbitrary units and r is the number of repetitions per set. In the larger aging study (shown in Fig. 9), muscle knee extensor strength and power were measured using a pneumatic resistance machine (Keiser Air300, Keiser Corporation, Fresno, CA, USA). Participants were instructed on proper performance of unilateral knee extension and performed 10 repetitions at minimal resistance as warm-up, followed by 3 sets of 5–10 repetitions at progressively increasing workloads. Participants then performed a series of single repetition attempts at increasing

workloads interspersed by 3 min of rest until they reached a level of resistance that could not be moved through the full range of motion. Peak power was measured at 70% of the individual 1-RM as participants performed the unilateral knee extension as quickly as possible through the full range of motion. Participants were allowed 3–5 attempts separated by 30 s. The highest measured power was recorded as the peak power.

Human immobilization study – Data came from a small pilot study involving healthy human subjects (5 females and 3 males, 50- to 63-years-old). The study was registered under Clinical Trial Number NCT04151901 and was performed at the University of Texas Medical Branch (UTMB). Subjects completed baseline unilateral knee extension strength testing in both legs via the 1-RM protocol described above. Five to ten days later, subjects returned, and baseline biopsies were taken from bilateral vastus lateralis muscles using the Bergström technique described above. Muscle samples were immediately frozen in liquid nitrogen and stored at -80 °C. Following bilateral muscle biopsies, a brace was placed on one leg of each subject to fix the knee in an extended position and prevent use of the quadriceps muscle, including the vastus lateralis. For the next 7 days, subjects wore the unilateral leg brace and ambulated with crutches or a walker. After 7 days of unilateral leg disuse, subjects returned and knee extensor 1RM and vastus lateralis biopsies were repeated bilaterally. Frozen muscle samples (~20-30 mg) were homogenized in TRIzol™, and total RNA was extracted using a guanidinium thiocyanate-phenol-chloroform based method (10). RNA concentration and purity was tested using a spectrophotometer (NanoDrop 2000; Thermo Scientific, Waltham, Massachusetts). Following RNA extraction, samples were sent to the UTMB Next Generation Sequencing (NGS) and Bioinformatics Core for library preparation and genome mapping, as described previously (11). Sequencing was performed using the Illumina NextSeq 550 High Output Flow Cell with the single-end 75 base protocol. Libraries were prepared using the SMART-3SEQ method (12) and NGS sequence reads were aligned using the Spliced Transcript Alignment to a Reference (STAR) program (13). FastQC was used for quality control. Once

aligned, the DESeq2 1.40.2 software package (R foundation) (14) was used to quantify *GADD45A* mRNA.

REFERENCES

1. Ebert SM, et al. Activating transcription factor 4 (ATF4) promotes skeletal muscle atrophy by forming a heterodimer with the transcriptional regulator C/EBPbeta. *J Biol Chem.* 2020;295(9):2787-803.
2. Ayers-Ringler JR, et al. Label-Free Proteomic Analysis of Protein Changes in the Striatum during Chronic Ethanol Use and Early Withdrawal. *Front Behav Neurosci.* 2016;10:46.
3. Murgia M, et al. Single muscle fiber proteomics reveals unexpected mitochondrial specialization. *EMBO Rep.* 2015;16(3):387-95.
4. Lanza IR, and Nair KS. Functional assessment of isolated mitochondria in vitro. *Methods Enzymol.* 2009;457:349-72.
5. Hart CR, et al. Attenuated activation of the unfolded protein response following exercise in skeletal muscle of older adults. *Aging (Albany NY).* 2019;11(18):7587-604.
6. Zhang X, et al. Preserved skeletal muscle oxidative capacity in older adults despite decreased cardiorespiratory fitness with ageing. *J Physiol.* 2021;599(14):3581-92.
7. Lalia AZ, et al. Effects of Dietary n-3 Fatty Acids on Hepatic and Peripheral Insulin Sensitivity in Insulin-Resistant Humans. *Diabetes Care.* 2015;38(7):1228-37.
8. Lalia AZ, Dasari S, Robinson MM, Abid H, Morse DM, Klaus KA, et al. Influence of omega-3 fatty acids on skeletal muscle protein metabolism and mitochondrial bioenergetics in older adults. *Aging (Albany NY).* 2017;9(4):1096-129.
9. Kalari KR, et al. MAP-RSeq: Mayo Analysis Pipeline for RNA sequencing. *BMC Bioinformatics.* 2014;15:224.
10. Chomczynski P, and Sacchi N. Single-step method of RNA isolation by acid guanidinium thiocyanate-phenol-chloroform extraction. *Anal Biochem.* 1987;162(1):156-9.

11. Ilinykh PA, et al. A single intranasal dose of human parainfluenza virus type 3-vectored vaccine induces effective antibody and memory T cell response in the lungs and protects hamsters against SARS-CoV-2. *NPJ Vaccines.* 2022;7(1):47.

12. Foley JW, et al. Gene expression profiling of single cells from archival tissue with laser-capture microdissection and Smart-3SEQ. *Genome Res.* 2019;29(11):1816-25.

13. Dobin A, et al. STAR: ultrafast universal RNA-seq aligner. *Bioinformatics.* 2013;29(1):15-21.

14. Love MI, Huber W, and Anders S. Moderated estimation of fold change and dispersion for RNA-seq data with DESeq2. *Genome Biol.* 2014;15(12):550.
